# Supplementary material for: And-1 coordinates with polymerase δ to regulate nucleotide excision repair and UVB-induced skin tumorigenesis
Source: Nat Commun. 2025 Oct 21;16:9313. doi: 10.1038/s41467-025-64380-4 (PMC12540712; doi:10.1038/s41467-025-64380-4)
Supplement: Supplementary file 1 — Supplementary Information [file 41467_2025_64380_MOESM1_ESM.pdf]

And-1 coordinates with polymerase δ to regulate nucleotide excision repair and UVB-induced skin tumorigenesis

Supplementary Information

This file includes:

Supplementary Figure 1. And-1 is a critical factor involved in repairing UVB-induced DNA lesions

Supplementary Figure 2. And-1 is crucial for the recruitment of p125 to chromatin in NER

Supplementary Figure 3. Phosphorylation of And-1 at T826 is required for its role in NER

Supplementary Figure 4. SepB and HMG domains of And-1 are required for repair synthesis

Supplementary Figure 5. DNA oligos and Purification of the FLAG-And-1 and V5-p125

Supplementary Figure 6. Generation and sequencing verification of $Wdhd1^{T819A}$ mice, along with histological analysis of epidermal thickness in mice

Supplementary Figure 7. Histological analysis of skin sections from *Wdhd1* WT mice and $Wdhd1^{T819A}$ mice

Supplementary Figure 8. Hematoxylin and Eosin (H&E) stain and immunohistochemical (IHC) stain for mice skin tumors

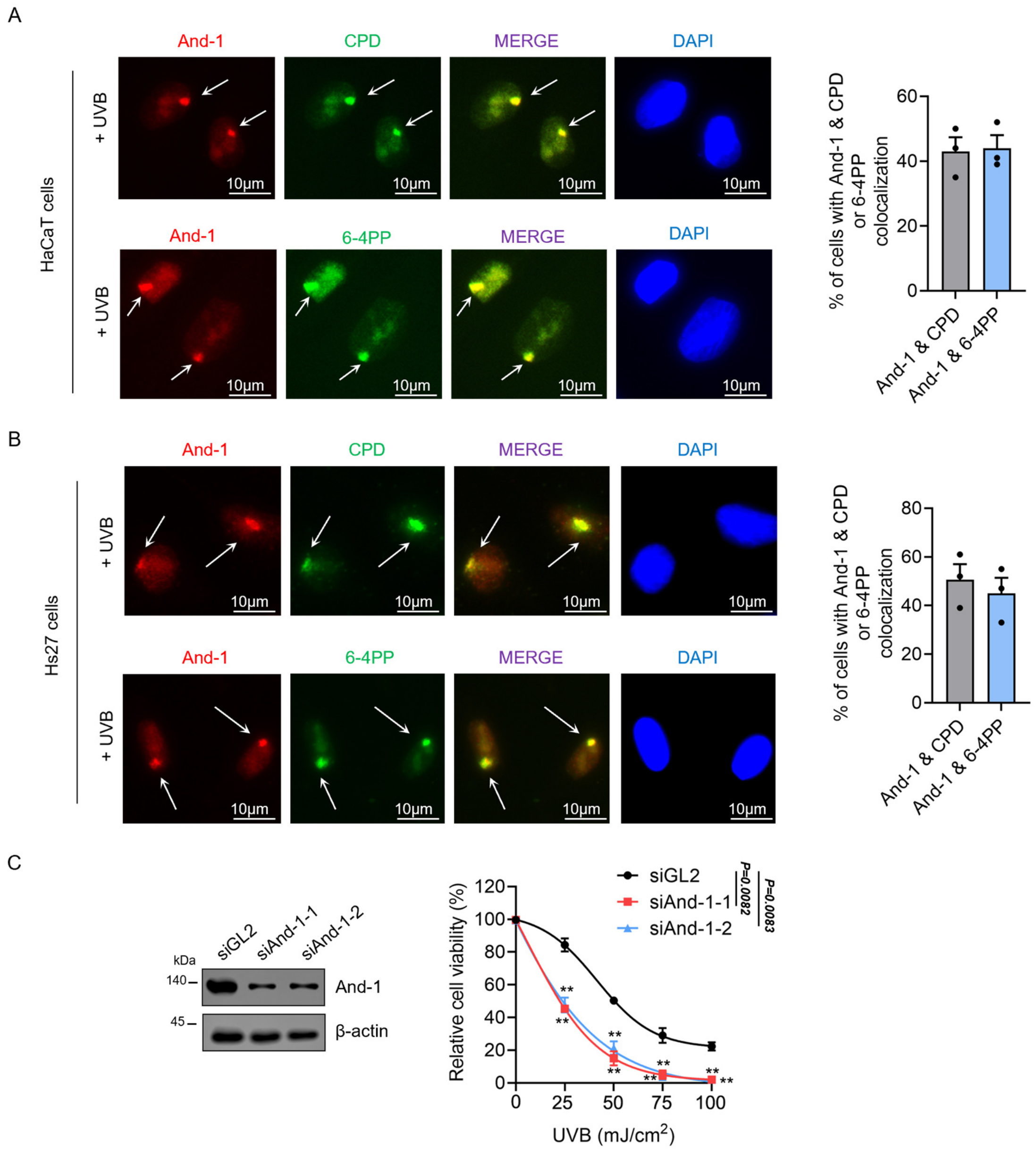

Supplementary Figure 1. And-1 is a crucial factor involved in repairing UVB-induced DNA lesions

(**A**) Left panel, immunofluorescence (IF) staining was used to examine the co-localization of And-1 with CPDs or 6-4PPs in response to UVB exposure in HaCaT cells. Cells were harvested 1 hour after UVB exposure at 75 mJ/cm². Right panel, quantification results of the percentage of cells with co-localization of And-1 with CPDs or 6-4PPs. Approximately 100 cells from three independent fields were counted and data was presented by mean ± SD. (**B**) Left panel, immunofluorescence was used to examine the co-localization of And-1 with CPDs or 6-4PPs in

response to UVB exposure in Hs27 cells. Cells were harvested 1 hour after UVB exposure at 75 mJ/cm^2. Right panel, quantification results of the percentage of cells with co-localization of And-1 with CPDs or 6-4PPs. Approximately 100 cells from three independent fields were counted and data was presented by mean ± SD. (**C**) HaCaT cells were transfected with indicated siRNAs, followed by exposure to UVB at indicated dosages one day after siRNA transfection. Cell viability was then measured 48 hours after UVB irradiation. Data shown in (C) were presented by mean ± SEM from three independent experiments. Statistic Analysis was performed by GraphPad Prism 9.0 using multiple two-tailed t-tests followed by Holm–Sidak correction. ** $P \leqslant 0.01$.

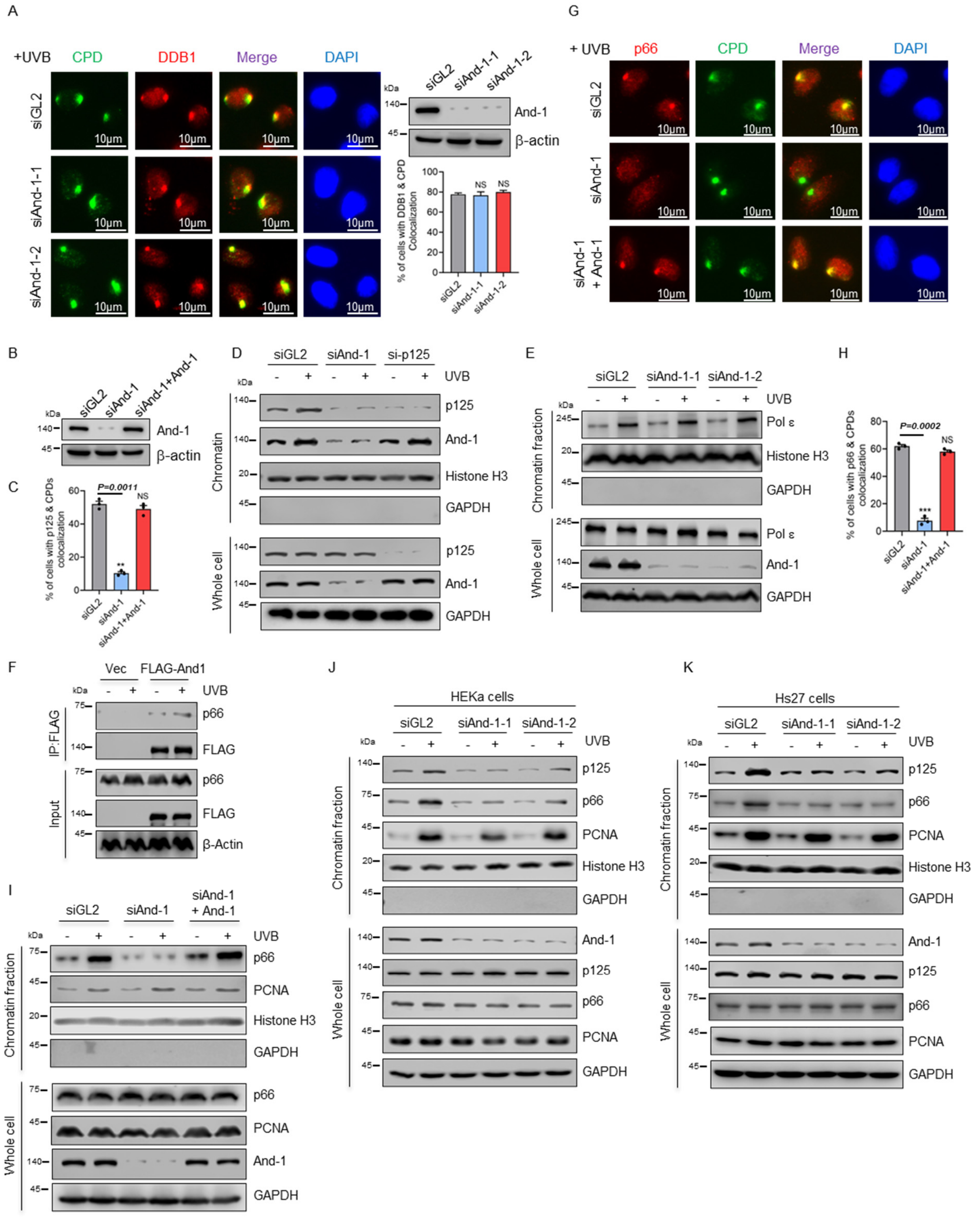
A
+UVB
CPD
DDB1
Merge
DAPI
siGL2
siAnd-1-1
siAnd-1-2
10μm
kDa
140
45
siGL2
siAnd-1-1
siAnd-1-2
And-1
β-actin
% of cells with DDB1 & CPD Colocalization
NS
NS
B
kDa
siGL2
siAnd-1
siAnd-1+And-1
And-1
β-actin
C
% of cells with p125 & CPDs colocalization
P=0.0011
NS
D
siGL2
siAnd-1
si-p125
UVB
Chromatin
Whole cell
p125
And-1
Histone H3
GAPDH
E
siGL2
siAnd-1-1
siAnd-1-2
UVB
Chromatin fraction
Whole cell
Pol ε
Histone H3
GAPDH
And-1
G
+ UVB
p66
CPD
Merge
DAPI
siGL2
siAnd-1
siAnd-1 + And-1
H
% of cells with p66 & CPDs colocalization
P=0.0002
NS
F
Vec
FLAG-And1
UVB
IP:FLAG
Input
p66
FLAG
β-Actin
I
siGL2
siAnd-1
siAnd-1 + And-1
UVB
Chromatin fraction
Whole cell
p66
PCNA
Histone H3
GAPDH
And-1
J
HEKa cells
siGL2
siAnd-1-1
siAnd-1-2
UVB
Chromatin fraction
Whole cell
p125
p66
PCNA
Histone H3
GAPDH
And-1
K
Hs27 cells
siGL2
siAnd-1-1
siAnd-1-2
UVB

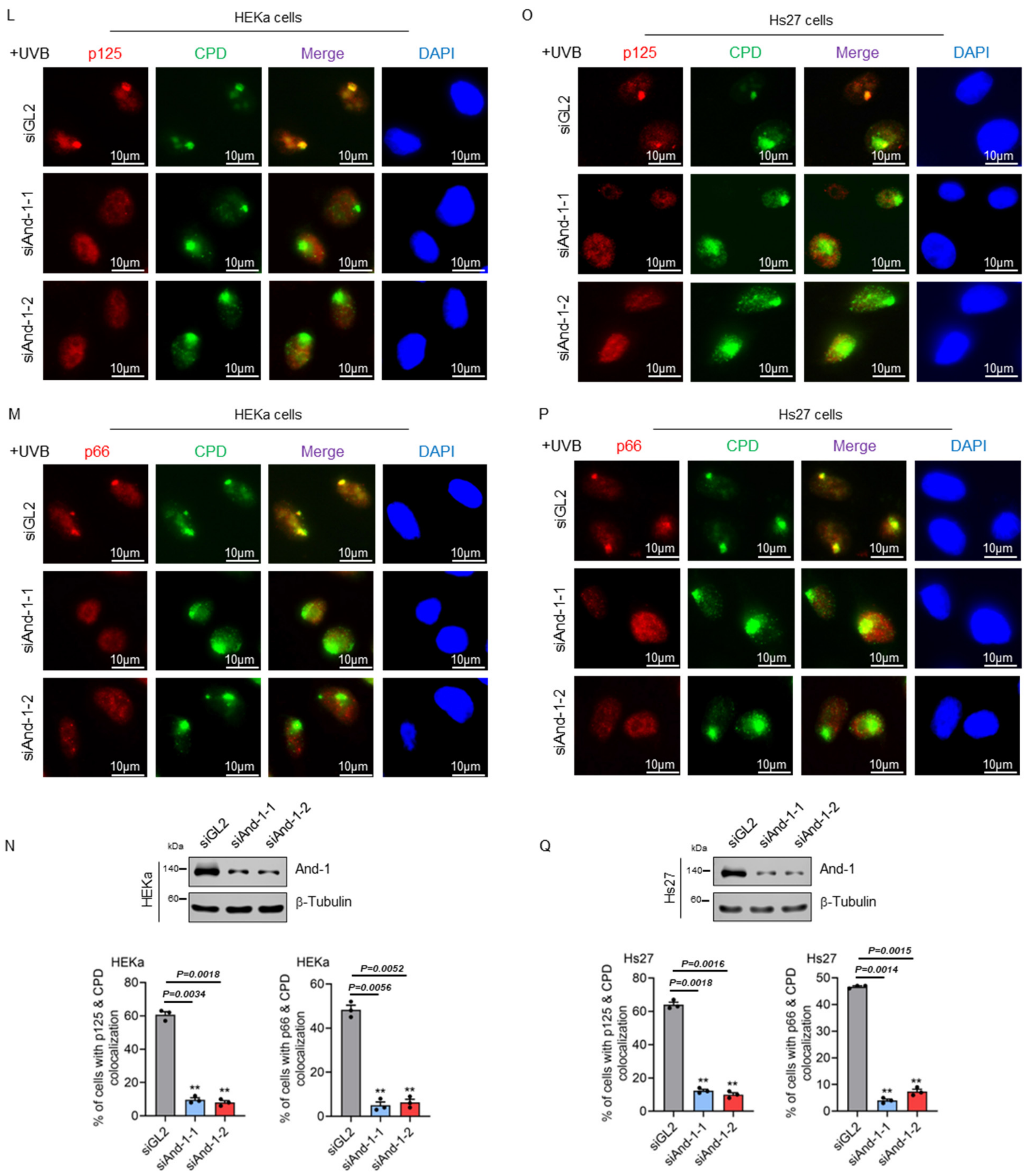

Supplementary Figure 2. And-1 is crucial for the recruitment of p125 to chromatin in NER
(**A**) HaCaT cells were transfected with indicated siRNA for 40 hours, then exposed to UVB at 75 mJ/cm². After one hour-repairing, IF staining then was performed to evaluate the co-localization

of DDB1 and CPDs. Approximately 100 cells from three independent fields were counted and data were presented by mean ± SD. (**B**) Cells shown in the Figure 2F were harvested and cell lysates were immunoblotted for indicated proteins. (**C**) Quantification results of the percentage of cells with co-localization of p125 with CPDs as shown in Figure 2F. Approximately 100 cells from three independent fields were counted and data were presented by mean ± SD. (**D-E**) HaCaT cells were transfected with indicated siRNAs for 40 hours, then exposed to UVB at 75 mJ/cm² and harvested 1 hour post-UVB irradiation. Chromatin fractions and whole-cell proteins were extracted and immunoblotted for indicated proteins. (**F**) HEK293T cells were transfected with the indicated plasmids for 40 hours, and then harvested 1 hour after UVB exposure at 100 mJ/cm². FLAG-IPs were performed in harvested cells, and IPs and inputs were then immunoblotted for the indicated proteins. (**G**) HaCaT cells were transfected with indicated siRNAs and plamids for 40 hours. Cells were then exposed to UVB at 75 mJ/cm² and then harvested for immunofluorescence staining 1 hour after UVB treatment. Immunofluorescence staining was performed to evaluate the co-localization of p66 and CPDs. (**H**) Quantification results of the percentage of cells with co-localization of p66 with CPDs. Approximately 100 cells from three independent fields were counted and data were presented by mean ± SD. (**I**) HaCaT cells were transfected with indicated siRNAs and plasmid for 40 hours, and then harvested 1 hour after exposed to UVB at 75 mJ/cm². Chromatin fractions and whole-cell proteins were extracted and immunoblotted for indicated proteins. (**J-K**) HEKa cells (J) and Hs27 cells (K) were transfected with indicated siRNA for 40 hours, and then harvested 1 hour after exposed to UVB at 75 mJ/cm². Chromatin fractions and whole-cell proteins were extracted and immunoblotted for indicated proteins. (**L-M**) HEKa cells were transfected with indicated siRNAs for 40 hours, and then harvested 1 hour post-UVB at 75 mJ/cm². Immunofluorescence staining was performed to examine the co-localization of p125 with CPDs (L) and p66 with CPDs (M). (**N**) Quantification results of the percentage of cells with co-localization of p125 or p66 with CPDs as shown in (L) and (M). Approximately 100 cells from three independent fields were counted and data were presented by mean ± SD. (**O-P**) Hs27 cells were transfected with indicated siRNAs for 40 hours, and then harvested 1 hour post-UVB at 75 mJ/cm². Immunofluorescence staining was then performed to examine the co-localization of p125 with CPDs (O) and p66 with CPDs (P). (**Q**) Quantification results of the percentage of cells with co-localization of p125 or p66 with CPDs as shown in (O) and (P). Approximately 100 cells from three independent fields were counted and data were presented by mean ± SD. For panels A, C, H,

N and Q, statistic analysis was performed using unpaired two-tailed t-tests in GraphPad Prism 9.0. $^{**}P \leqslant 0.01$, $^{***}P \leqslant 0.001$, “NS” indicates no significant difference.

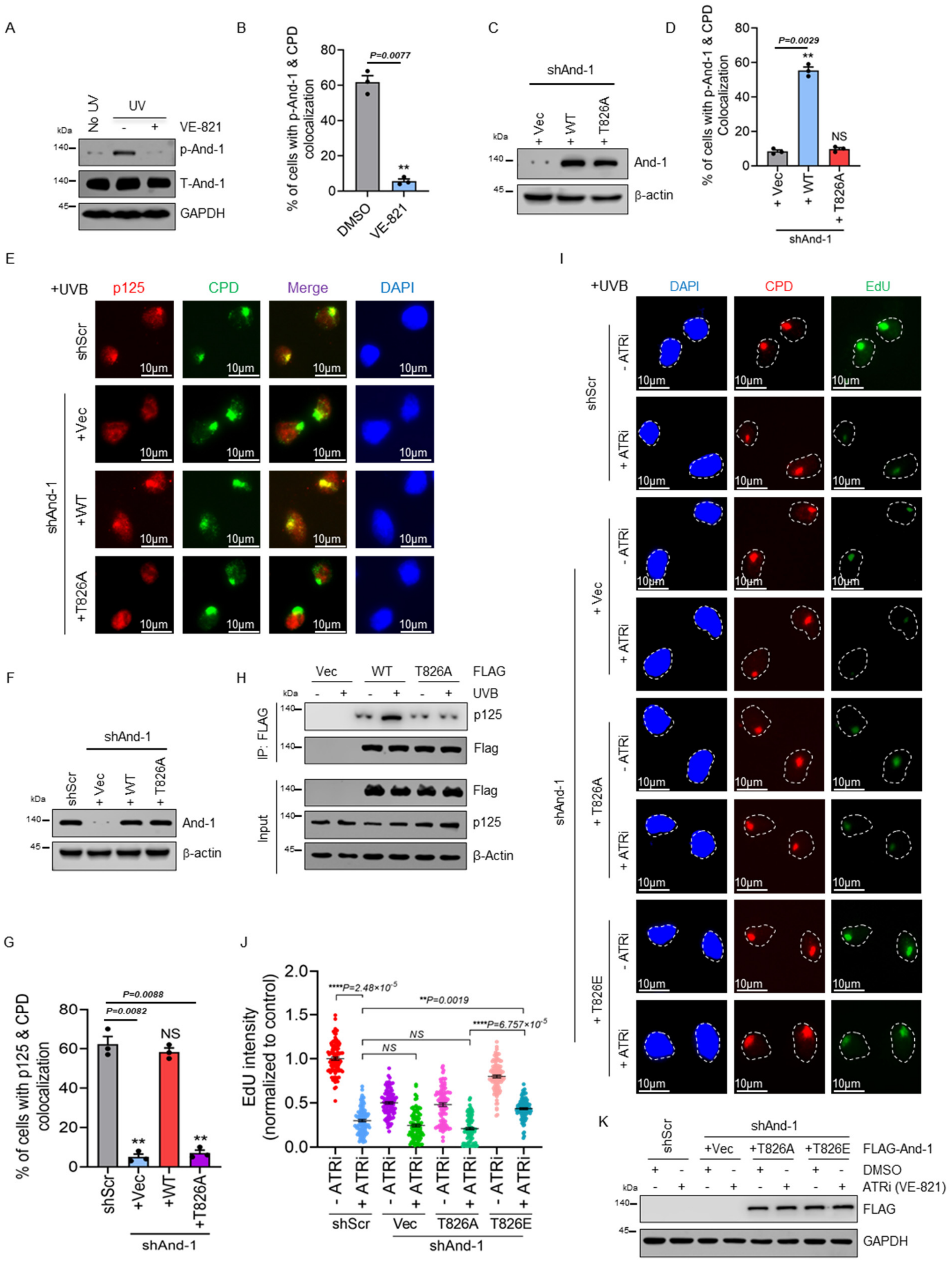

A
No UV
UV
-
+
VE-821
kDa
140
p-And-1
140
T-And-1
45
GAPDH
B
P=0.0077
% of cells with p-And-1 & CPD colocalization
80
60
40
20
0
**
DMSO
VE-821
C
shAnd-1
+ Vec
+ WT
+ T826A
kDa
140
And-1
45
β-actin
D
P=0.0029
**
NS
% of cells with p-And-1 & CPD Colocalization
80
60
40
20
0
+ Vec
+ WT
+ T826A
shAnd-1
E
+UVB
p125
CPD
Merge
DAPI
shScr
+Vec
+WT
+T826A
shAnd-1
10μm
F
shAnd-1
shScr
+ Vec
+ WT
+ T826A
kDa
140
And-1
45
β-actin
G
P=0.0088
P=0.0082
NS
**
**
% of cells with p125 & CPD colocalization
80
60
40
20
0
shScr
+Vec
+WT
+T826A
shAnd-1
H
Vec
WT
T826A
FLAG
UVB
kDa
-
+
IP: FLAG
140
p125
140
Flag
Input
140
Flag
140
p125
45
β-Actin
I
+UVB
DAPI
CPD
EdU
shScr
- ATRi
+ ATRi
+ Vec
+ T826A
+ T826E
shAnd-1
10μm
J
****P=2.48×10^-5
**P=0.0019
NS
NS
****P=6.757×10^-5
EdU intensity (normalized to control)
2.0
1.5
1.0
0.5
0.0
- ATRi
+ ATRi
shScr
Vec
T826A
T826E
shAnd-1
K
shScr
shAnd-1
+Vec
+T826A
+T826E
FLAG-And-1
DMSO
ATRi (VE-821)
kDa
140
FLAG
45
GAPDH

Supplementary Figure 3. Phosphorylation of And-1 at T826 is required for its role in recruiting p125

(**A**) HaCaT cells were pretreated with or without 5μM ATRi (VE-821) for 6 hours, and then exposed to UVB at 75 mJ/cm² and harvested 1 hour later. Cell lysates were immunoblotted for indicated proteins. (**B**) Quantification results of the co-localization of p-And-1 and CPDs as shown in Figure 3A. Approximately 100 cells from three independent fields were counted and data were presented by mean ± SD. (**C**) And-1-depleted HaCaT cells were transfected with the indicated plasmids for 40 hours, and then exposed to UVB at 75 mJ/cm² and harvested 1 hour later. Cell lysates were immunoblotted for indicated proteins. (**D**) Quantification results of the co-localization of p-And-1 and CPDs as shown in Figure 3B. Approximately 100 cells from three independent fields were counted and data were presented by mean ± SD. (**E**) And-1-depleted HaCaT cells were transfected with the indicated plasmids for 40 hours, cells were then harvested for IF assay to examine the co-localization of CPDs with p125 one hour post-UVB irradiation at 75 mJ/cm^2. (**F**) Cells shown in (E) were harvested before UVB treatment, Western blotting was performed to measure the indicated proteins. (**G**) Quantification results of the co-localization of p125 and CPDs as shown in (E). Approximately 100 cells from three independent fields were counted and data were presented by mean ± SD. (**H**) HEK293T cells were transfected with the indicated plasmids for 40 hours, and cells were then harvested 1 hour after UVB exposure at 100 mJ/cm². FLAG-IP was performed in harvested cells, and IPs were then immunoblotted for indicated proteins. (**I**) And-1 depleted HaCaT cells were transfected with the indicated plasmids for 40 hours, followed by pretreated with or without 5 μM ATRi (VE-821) for 6 hours. Cells were then exposed to UVB at 75 mJ/cm² and harvested 2 hour post UVB treatment to to assess Edu incorporation efficiency by unscheduled DNA synthesis (UDS) assay. DNA damage was indicated by CPD staining. (**J**) Quantification results of EdU levels as shown in (I), normalized to control cells. Approximately 100 cells were counted and data were presented by mean ± SD. (**K**) Cells shown in (I) were harvested before UVB treatment and immunoblotted for indicated proteins. For panels B, D, G and J, statistic analysis was performed using upaired two-tailed t-tests in GraphPad Prism 9.0. $^{**}P \leqslant 0.01$, $^{***}P \leqslant 0.001$, "*NS*" indicates no significant difference.

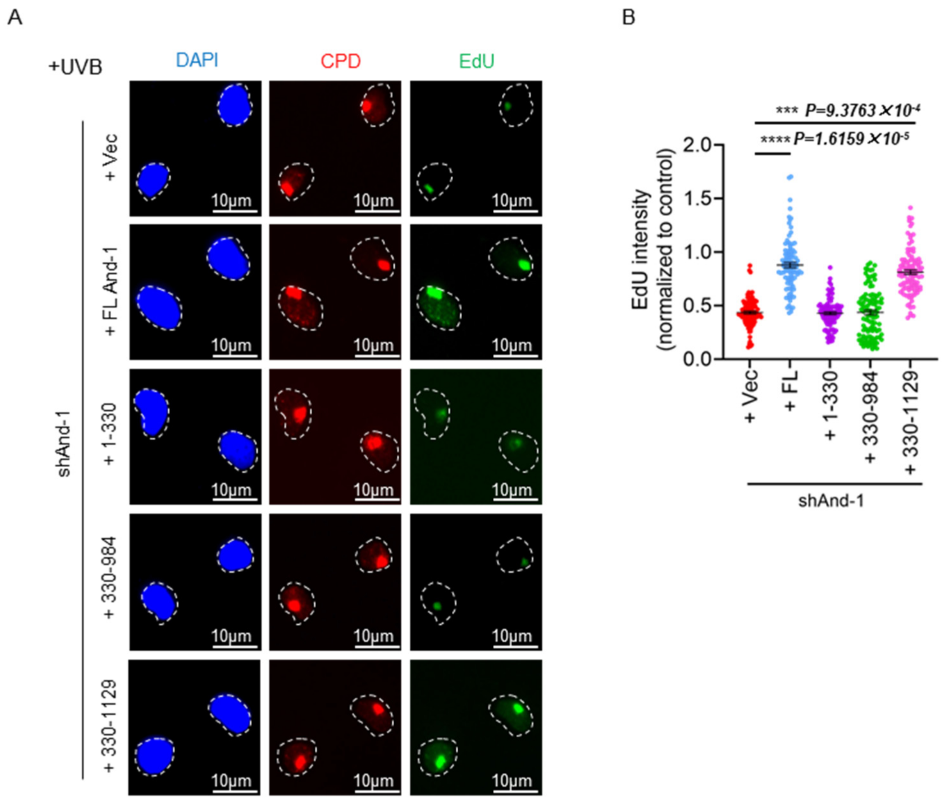

Supplementary Figure 4. SepB and HMG domains of And-1 are required for NER repair

(**A**) And-1-depleted HaCaT cells were transfected with the indicated And-1 mutant plasmids for 40 hours, followed by exposure to UVB at 75 mJ/cm² and harvested 2 hour-post UVB irradiation for UDS assay to assess Edu incorporation efficiency. DNA damage was indicated by CPD staining. (**B**) Quantification results of EdU levels, normalized to control cells. Approximately 100 cells from three independent fields were counted and data were presented by mean ± SD. Statistic analysis was performed using unpaired two-tailed t-tests in GraphPad Prism 9.0. ***$P \leqslant 0.001$.

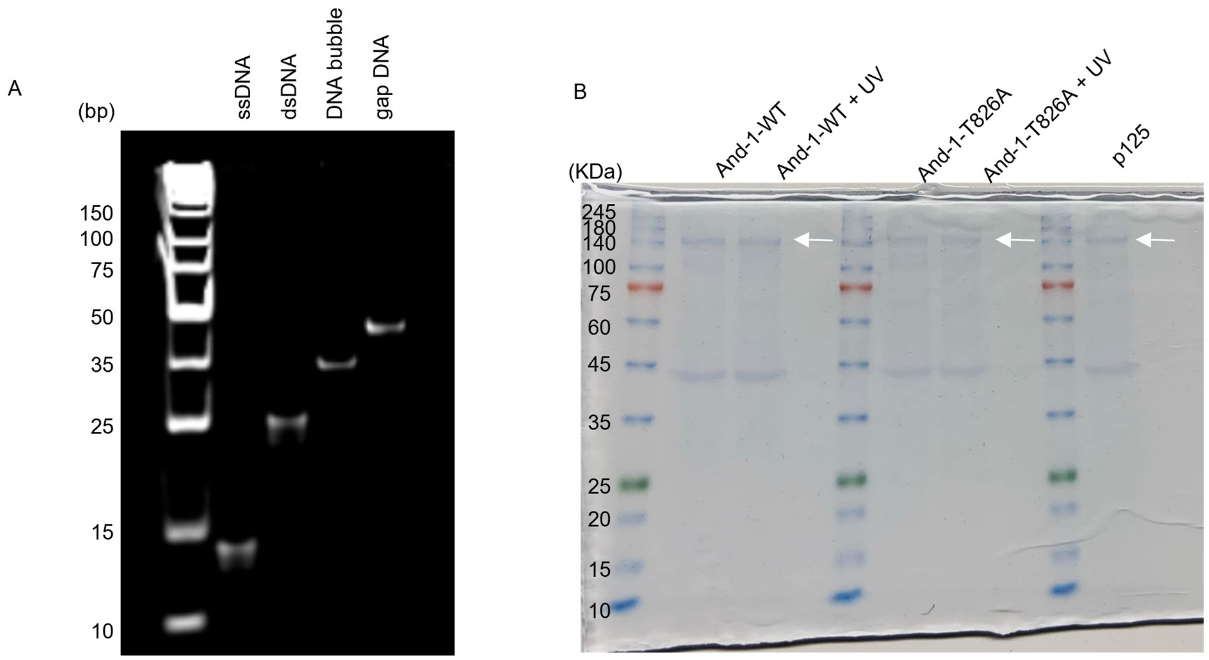

Supplementary Figure 5. DNA oligos and purification of the FLAG-And-1 and V5-p125

(**A**) Purified DNA structures ssDNA, dsDNA, DNA bubble, and gap DNA were resolved on a 20% native polyacrylamide gel. (**B**) Coomassie blue staining of purified protein of FLAG-And-1-WT, FLAG-And-1-T826A with or without UVB treatment at 100 mJ/cm² , as well as the V5-p125 from HEK293T cells. White arrows indiated purified And-1 proteins or p125 protein.

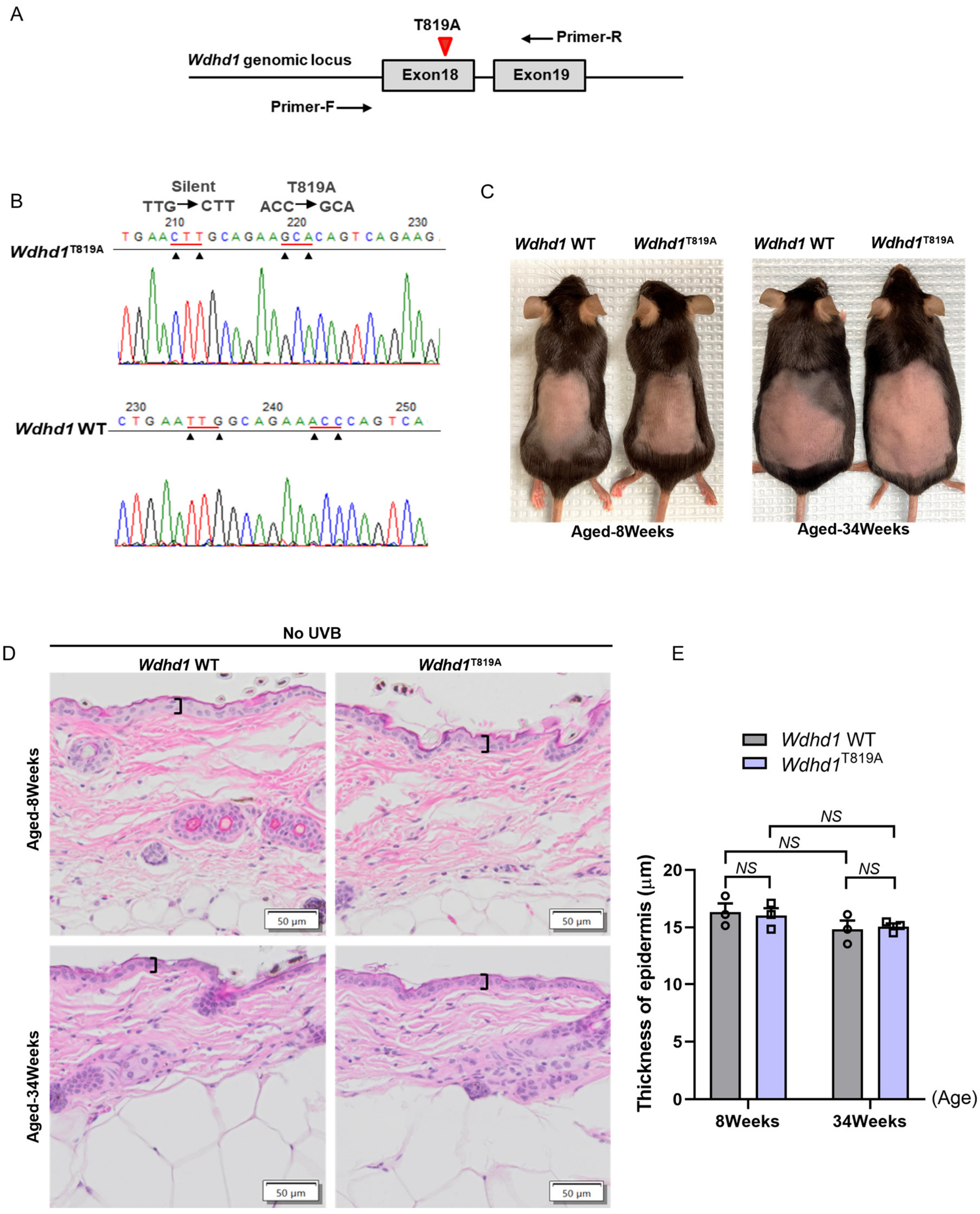

Supplementary Figure 6. Generation and sequencing verification of *Wdhd1^{T819A}* mice, along with histological analysis of epidermal thickness in mice.

(**A**) Schematic representation of CRISPR/Cas9-mediated generation of the *Wdhd1*$^{\mathrm{T819A}}$ allele within the exon 18, along with the locations of PCR primers (shown in the Methods section) for sequencing. (**B**) Genotyping sequencing results confirm the generation of *Wdhd1*$^{\mathrm{T819A}}$ mice

through an ACC-to-GCA mutation at the T819 site. A silent mutation (TTG to CTT) was also introduced at the T816 site (upper panel). Representative sequencing results from *Wdhd1* WT mice are shown in the lower panel. (**C**) The representation images of skin appearance of both *Wdhd1* WT and *Wdhd1*^{T819A}mice at 8 weeks and 34 weeks. (**D**) The representation images of epidermal thickness in both *Wdhd1* WT and *Wdhd1*^{T819A} mice at 8 weeks and 34 weeks by H&E staining, respectively. (**E**) The quantification results of epidermal thickness as shown in (D). Data was shown as mean ± SEM. Statistical analysis was performed using two-way ANOVA in GraphPad Prism 9.0, with "*NS*" indicating no significant difference.

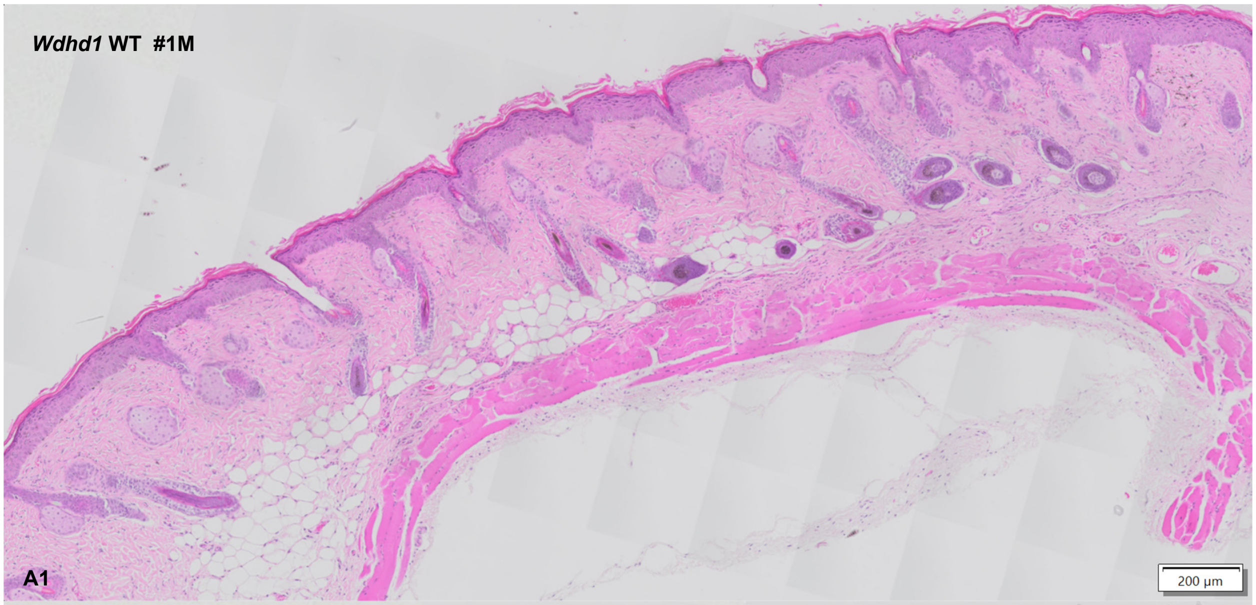
Wdhd1 WT #1M
A1
200 µm

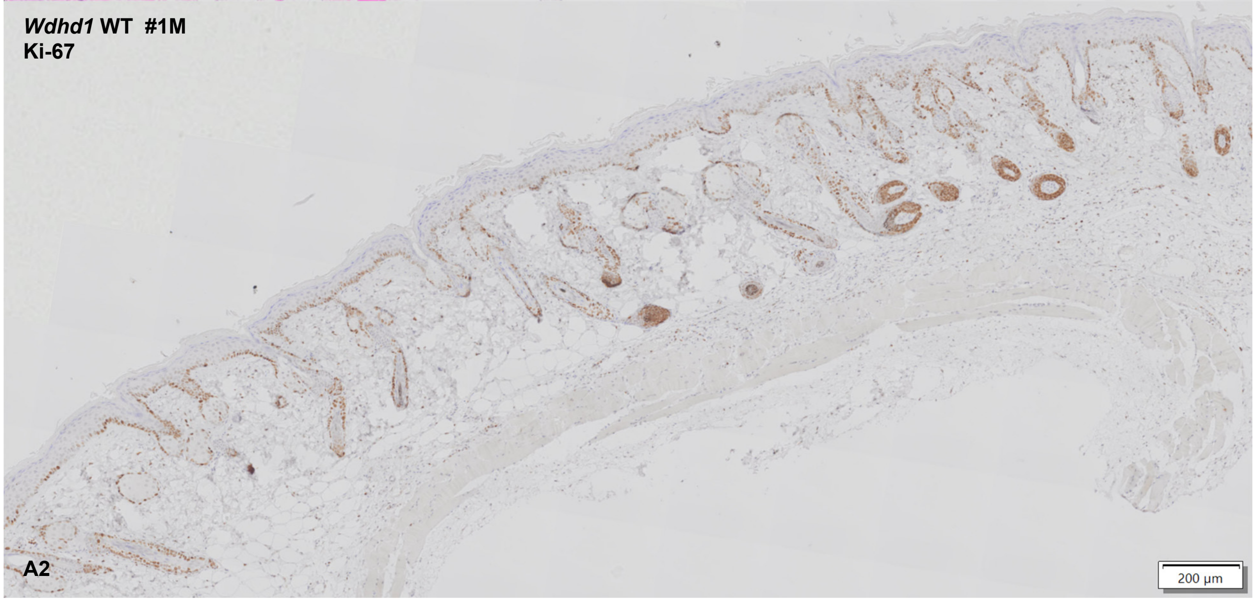
Wdhd1 WT #1M
Ki-67
A2
200 µm

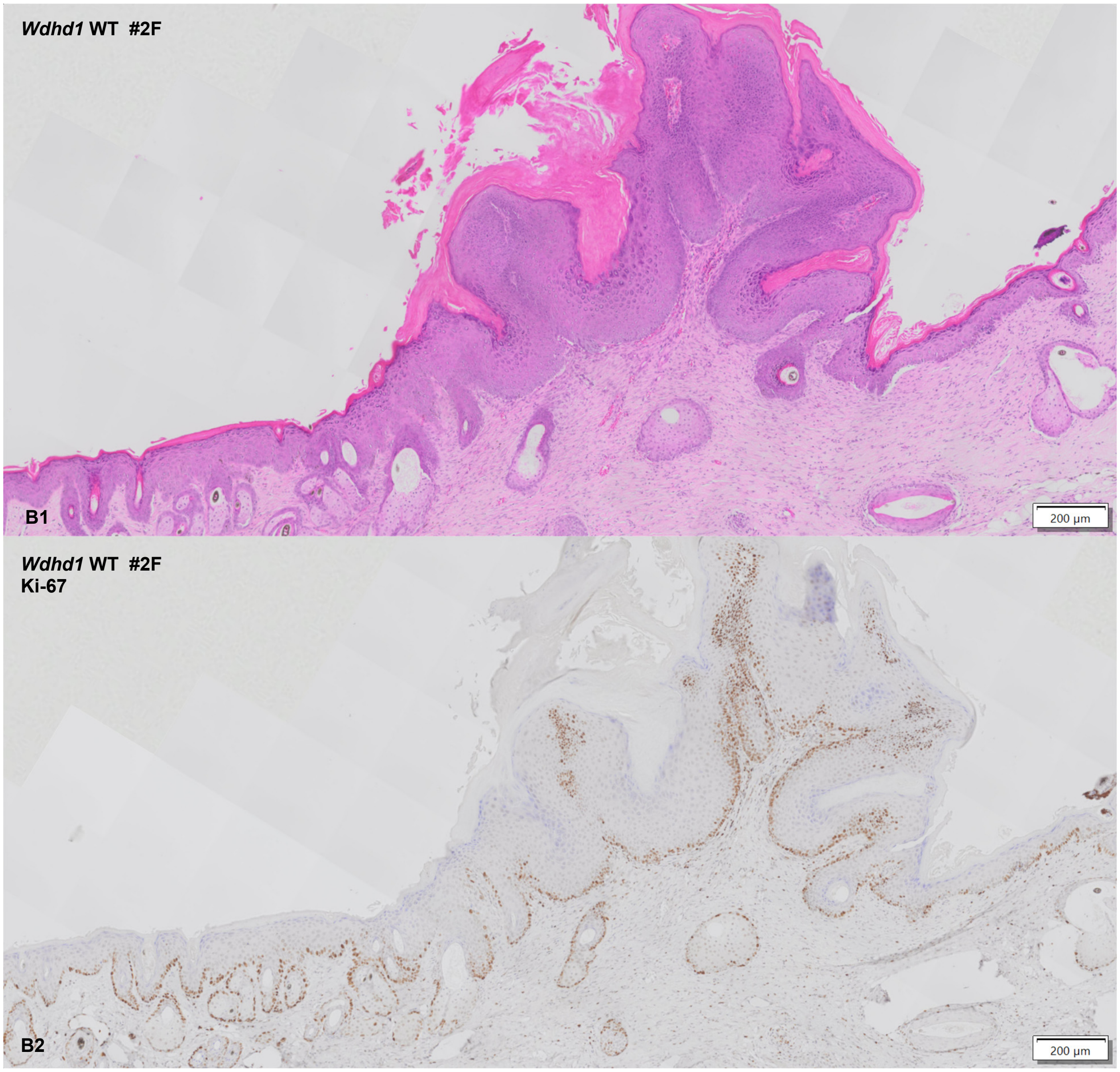
Wdhd1 WT #2F
B1
200 µm
Wdhd1 WT #2F
Ki-67
B2
200 µm

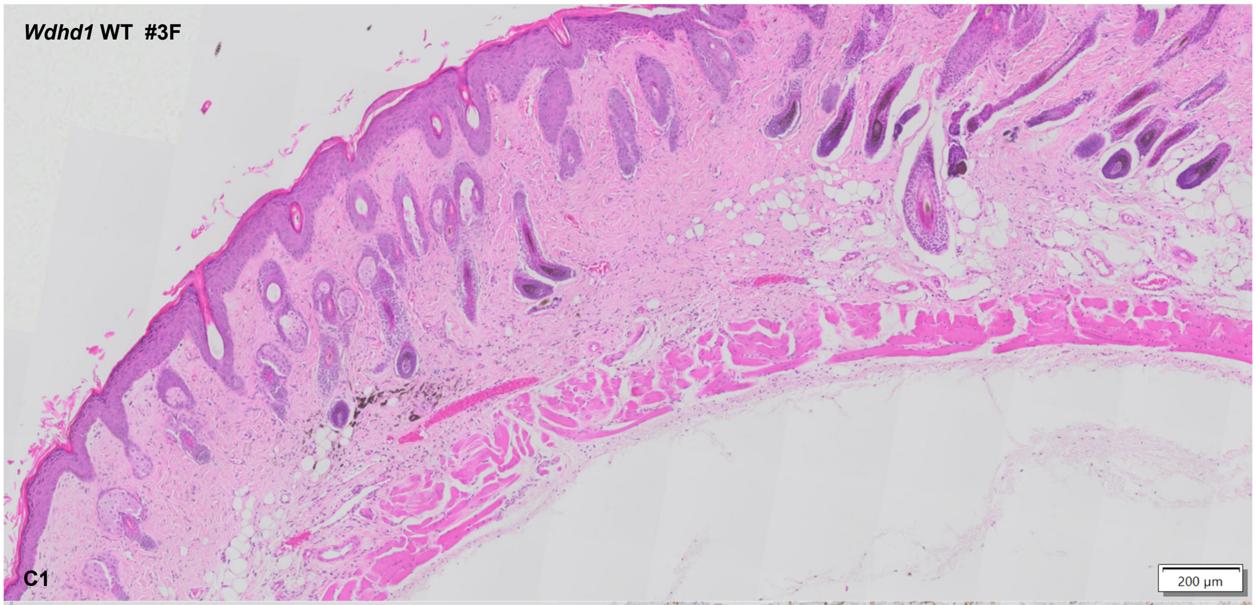

Wdhd1 WT #3F
C1
200 µm

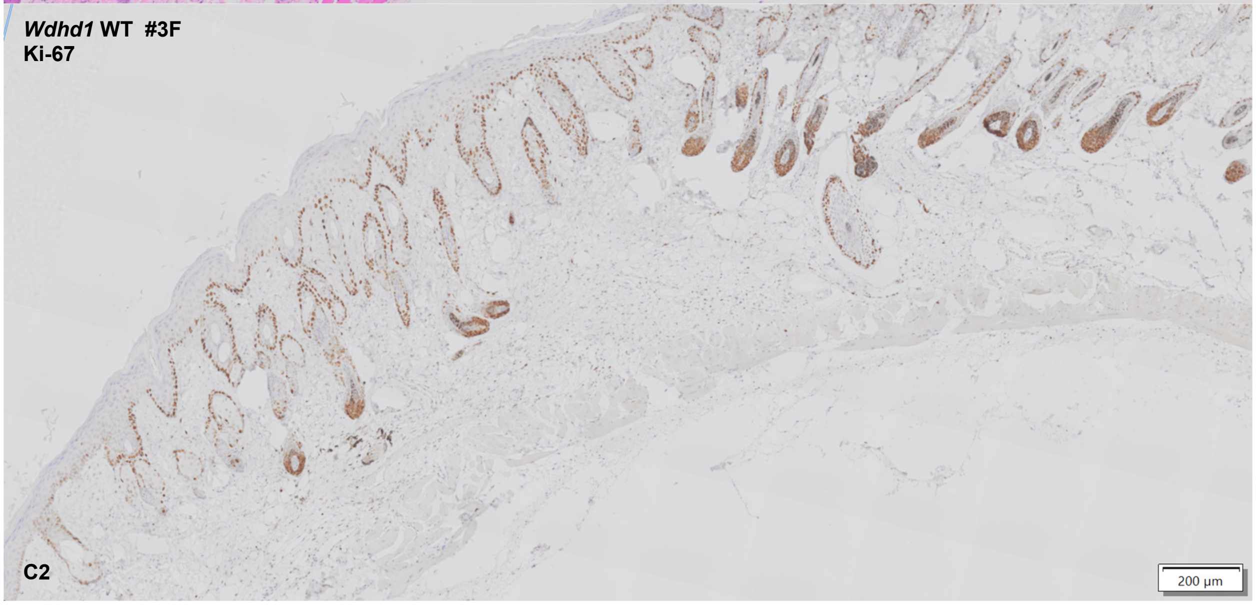

Wdhd1 WT #3F
Ki-67
C2
200 µm

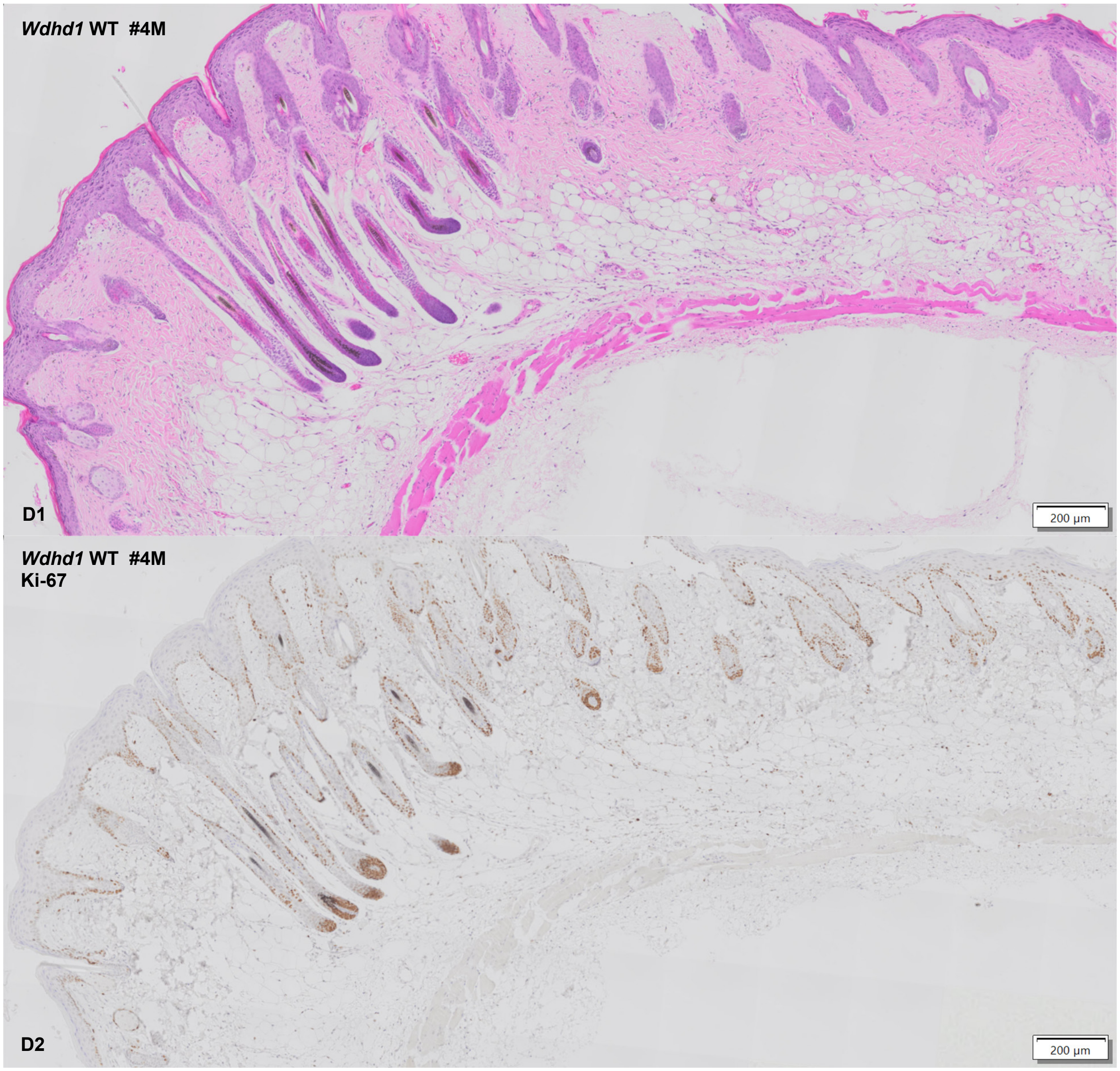
Wdhd1 WT #4M
D1
200 µm
Wdhd1 WT #4M
Ki-67
D2
200 µm

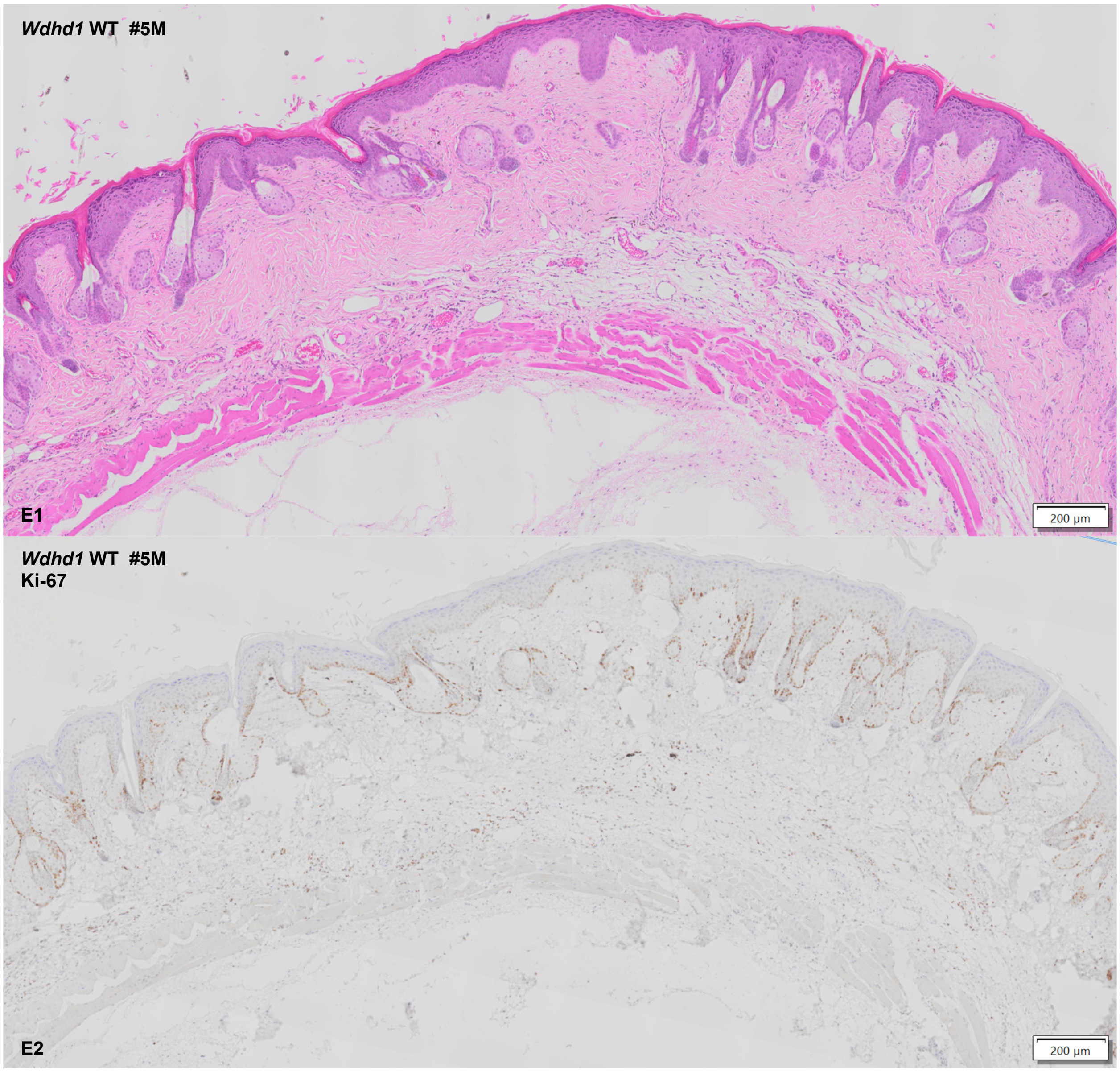
Wdhd1 WT #5M
E1
200 µm
Wdhd1 WT #5M
Ki-67
E2
200 µm

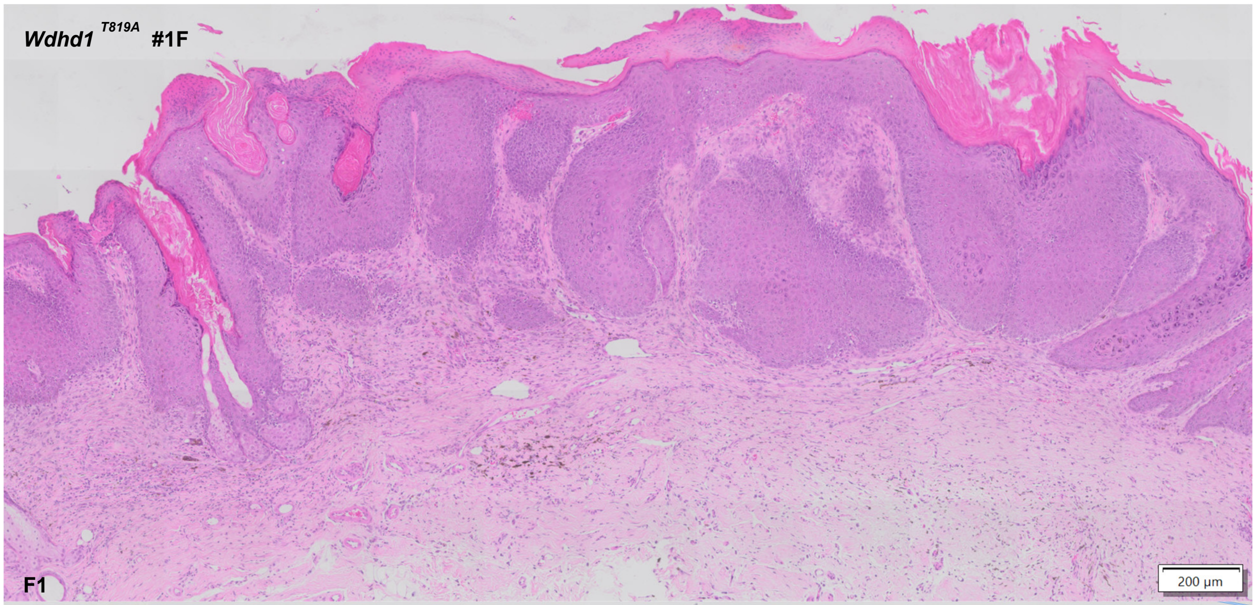

Wdhd1 *T819A* #1F
F1
200 µm

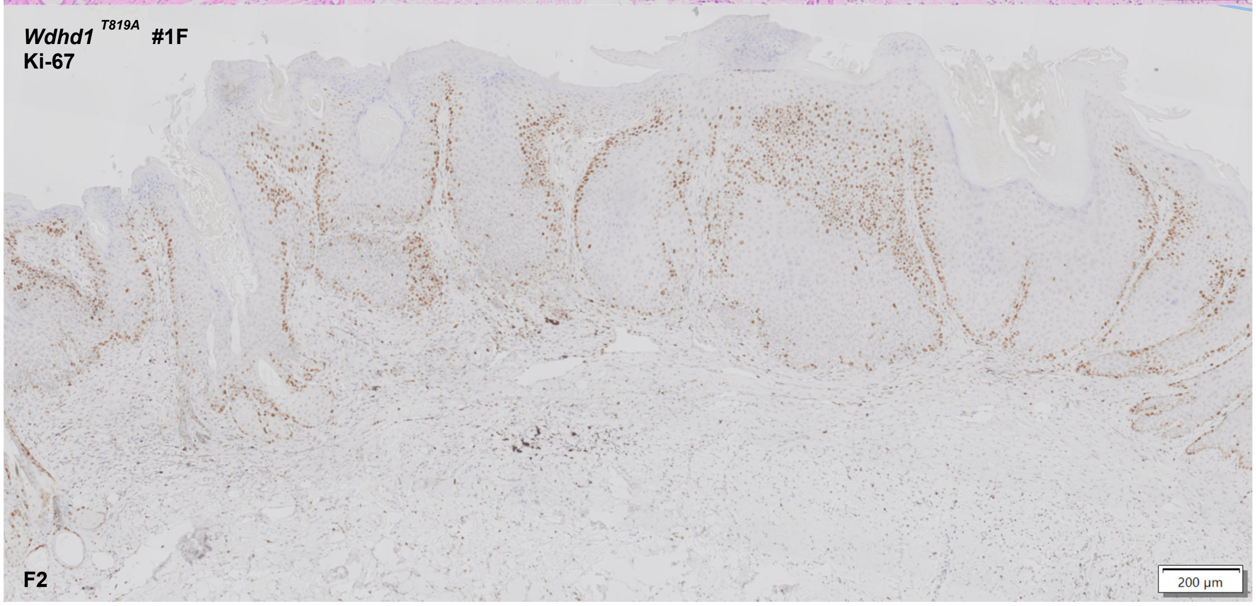

Wdhd1 *T819A* #1F
Ki-67
F2
200 µm

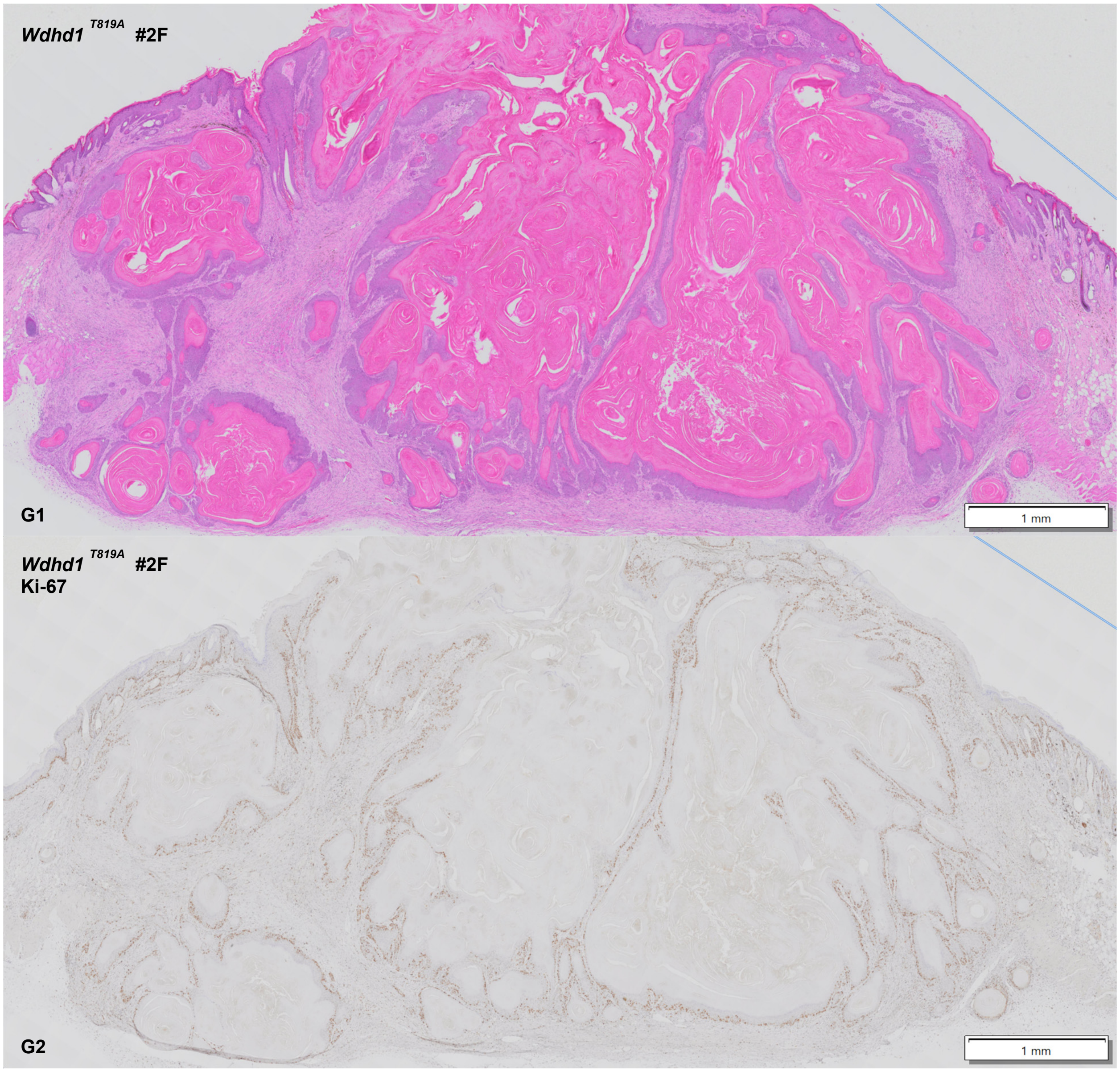
Wdhd1 T819A #2F
G1
1 mm
Wdhd1 T819A #2F
Ki-67
G2
1 mm

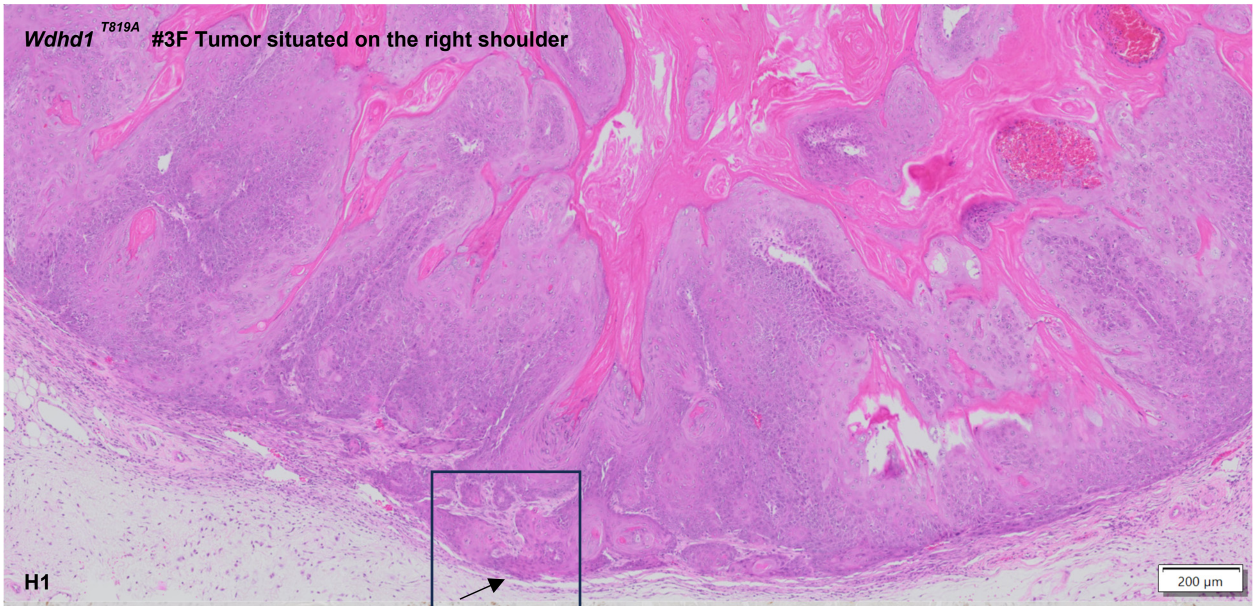

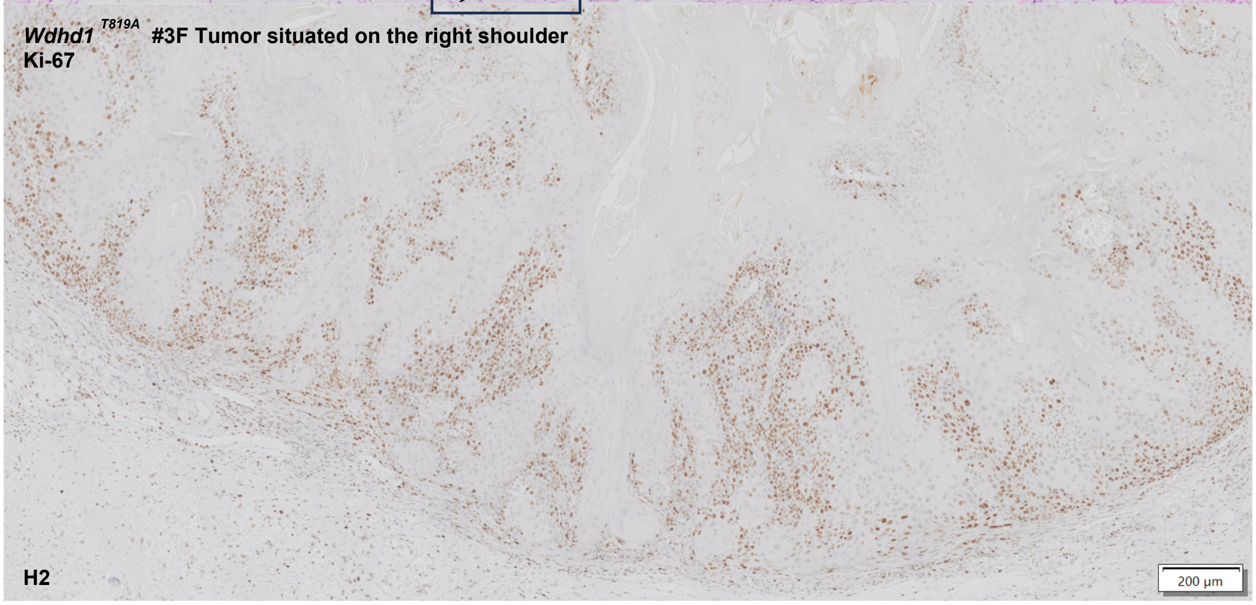

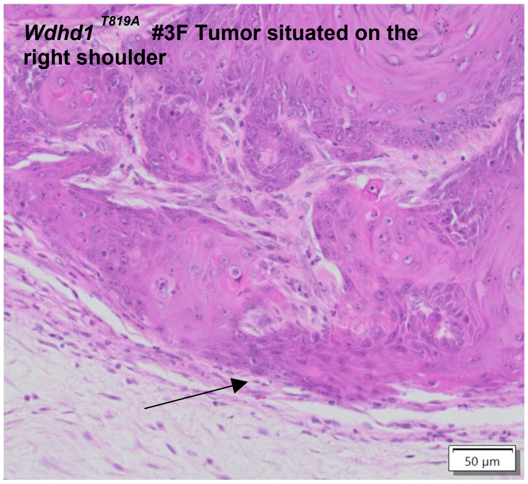

*This is the zoomed-in images of the square region indicated in the top panel. Black arrow indicated the micro invasion areas of this tumor.

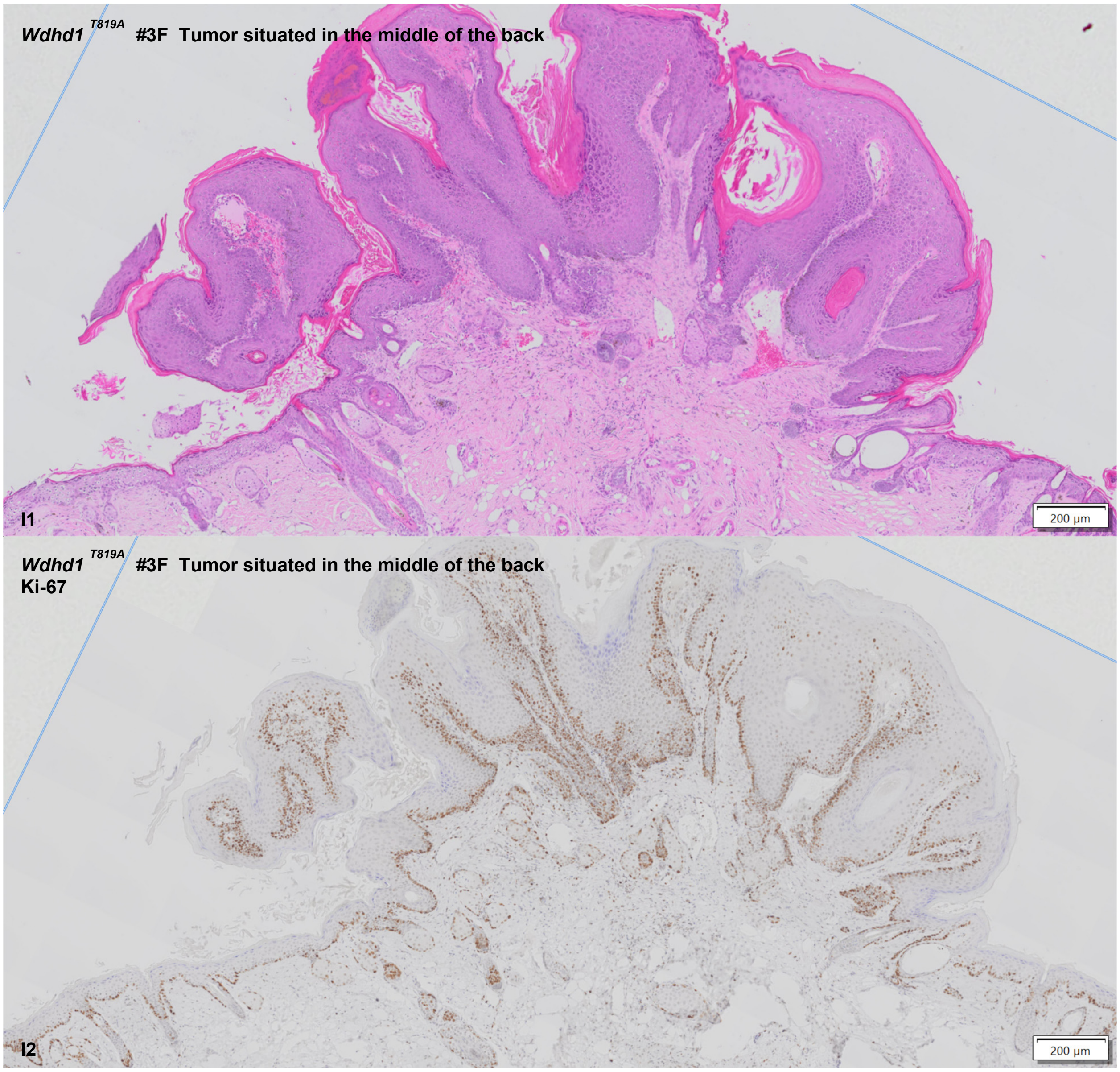
Wdhd1 ^{T819A} #3F Tumor situated in the middle of the back
I1
200 µm
Wdhd1 ^{T819A} #3F Tumor situated in the middle of the back
Ki-67
I2
200 µm

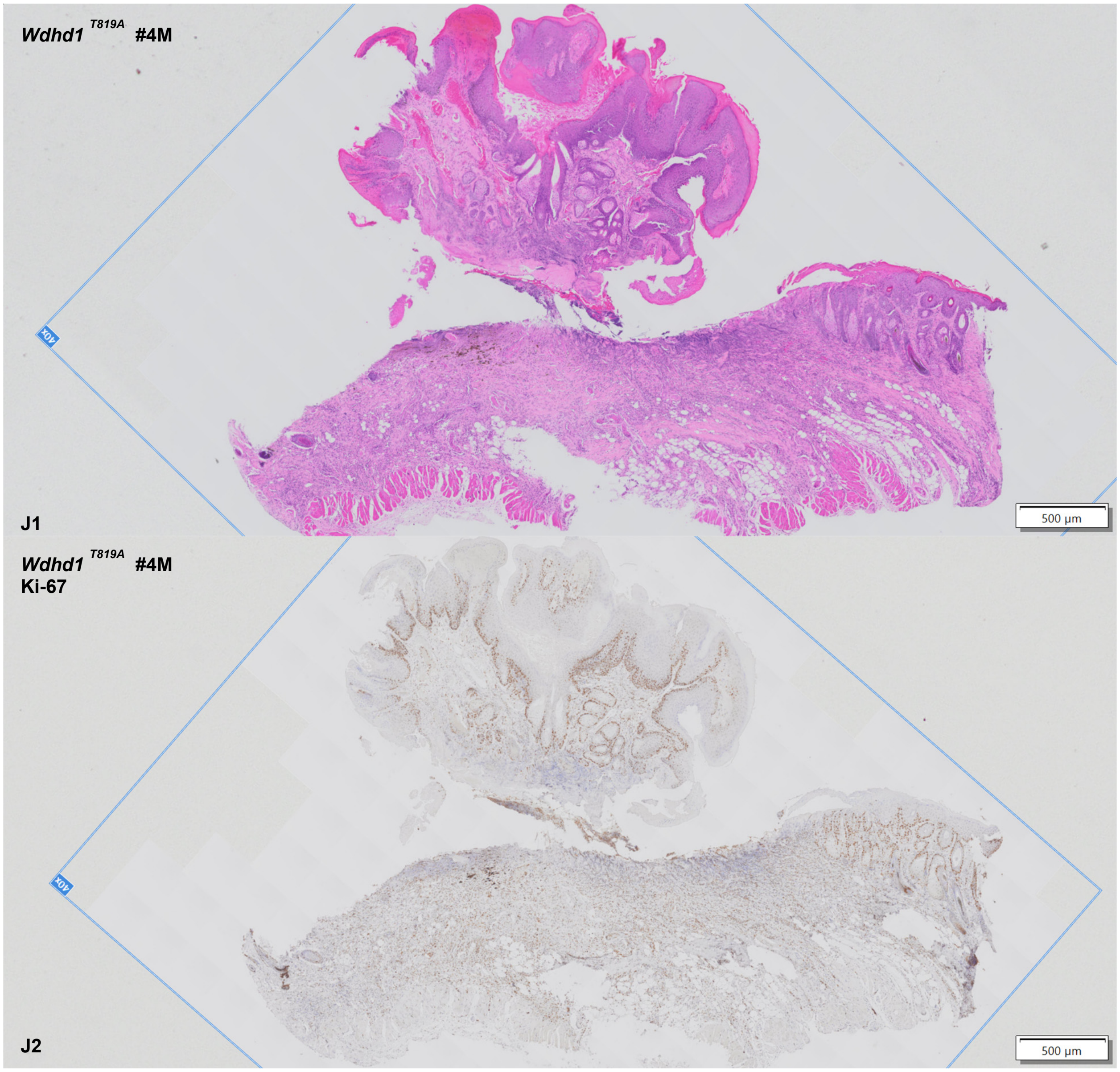
Wdhd1 T819A #4M
J1
500 µm
Wdhd1 T819A #4M
Ki-67
J2
500 µm

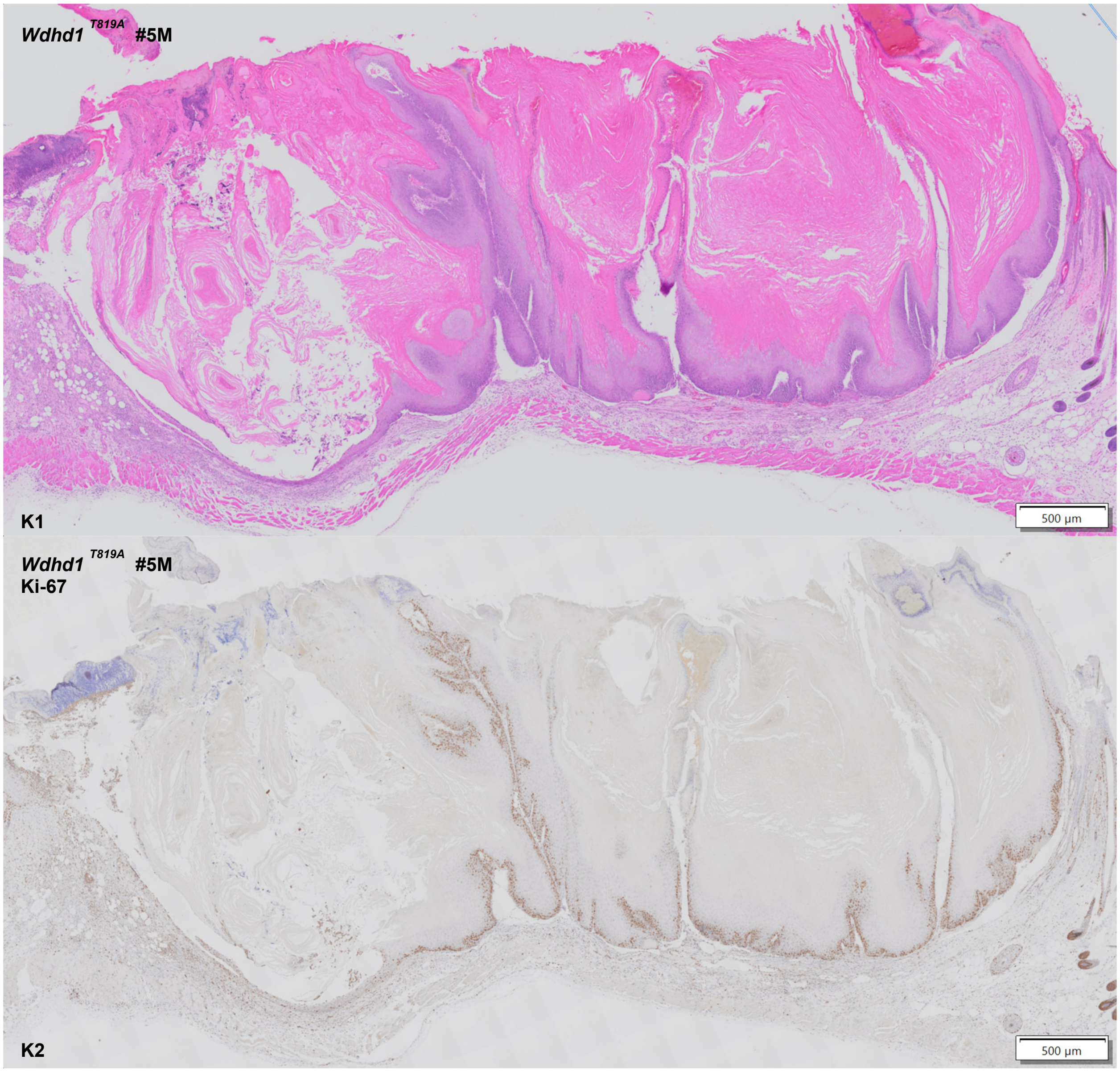

Supplementary Figure 7. Histological analysis of skin sections from *Wdhd1* WT mice and *Wdhd1*^{T819A} mice.

The representation images of mice skin or keratoacanthoma sections from *Wdhd1* WT (**A1-E2**) and *Wdhd1*^{T819A} mice (**F1-K2**), stained with H&E and Ki-67 immunohistochemistry, respectively. Scale bars of 50μm, 200 μm, 500 μm, and 1 mm were indicated in the corresponding images, as full scans were performed on tumors or skin tissues of varying sizes. Mice are denoted as male (M) or female (F).

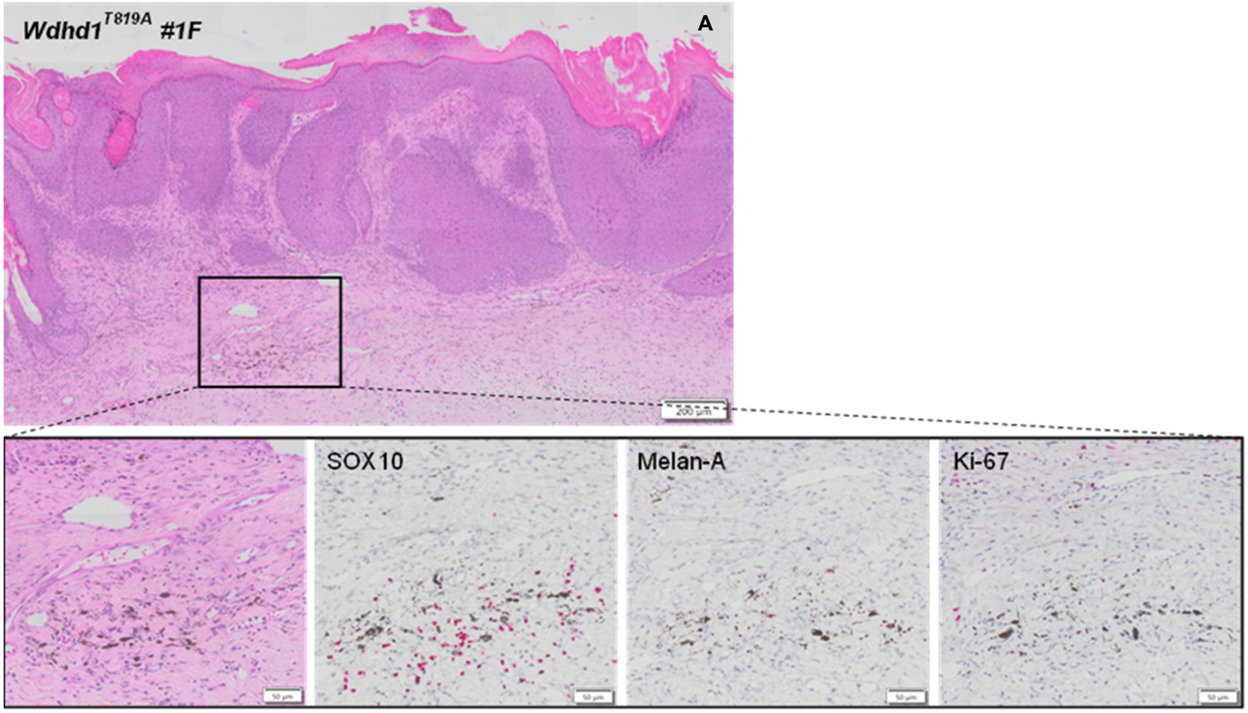

Wdhd1^{T819A} #1F
A
200 μm
SOX10
Melan-A
Ki-67

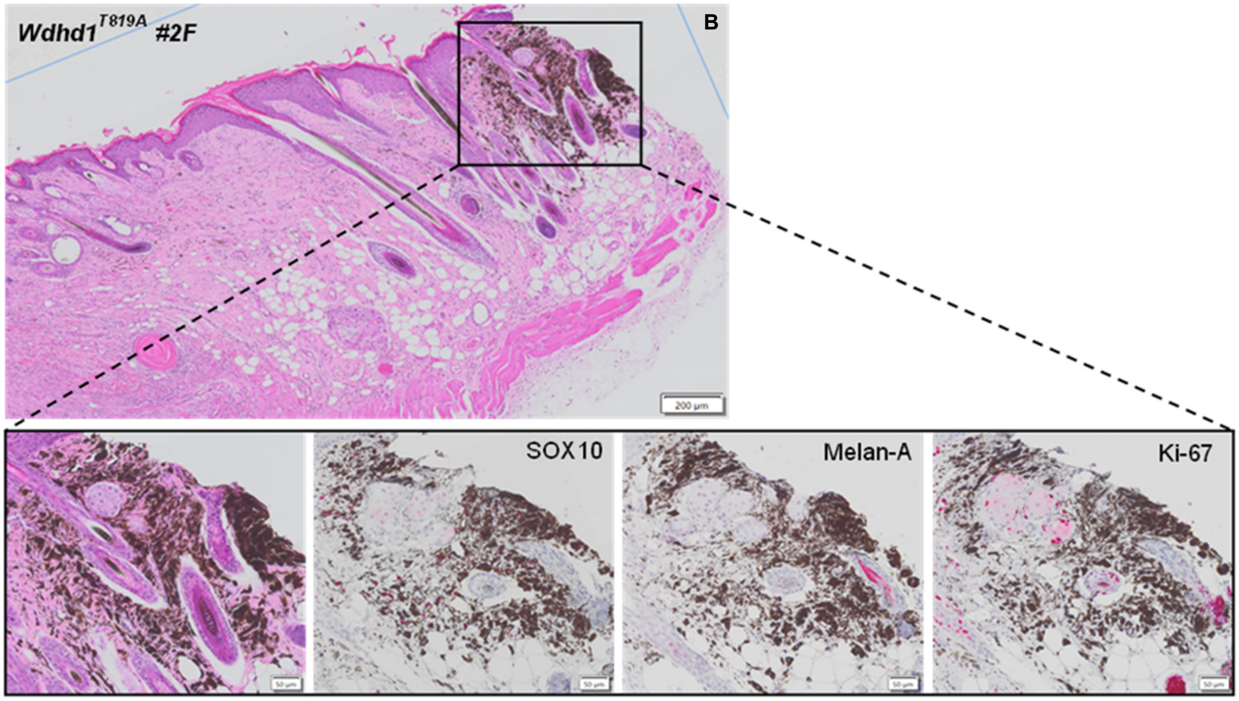

Wdhd1^{T819A} #2F
B
200 μm
SOX10
Melan-A
Ki-67

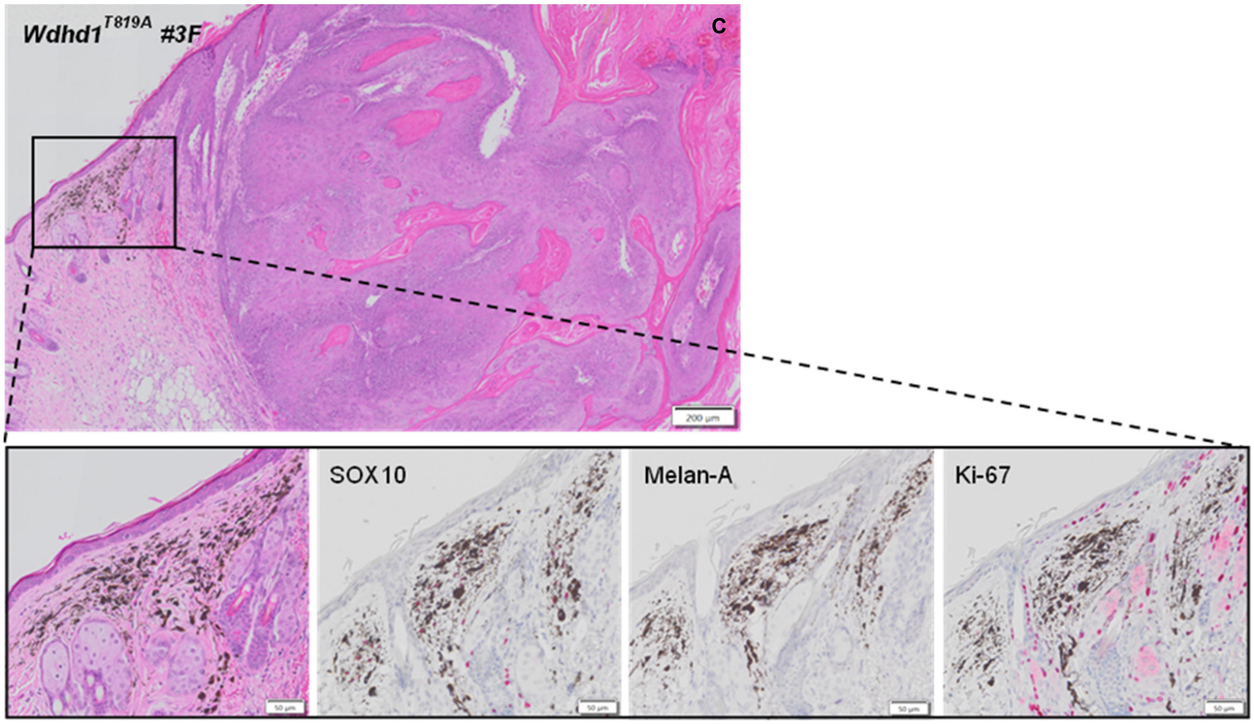
Wdhd1^{T819A} #3F
C
200 µm
SOX10
Melan-A
Ki-67
50 µm

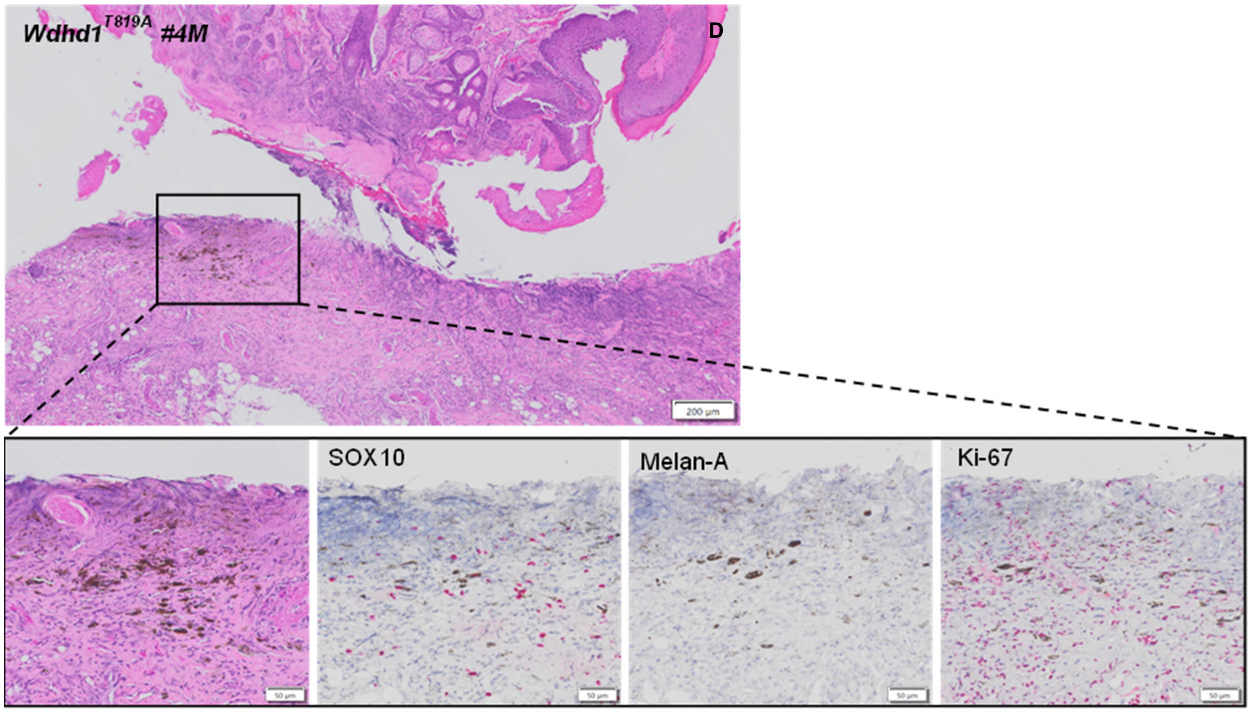
Wdhd1^{T819A} #4M
D
200 µm
SOX10
Melan-A
Ki-67
50 µm

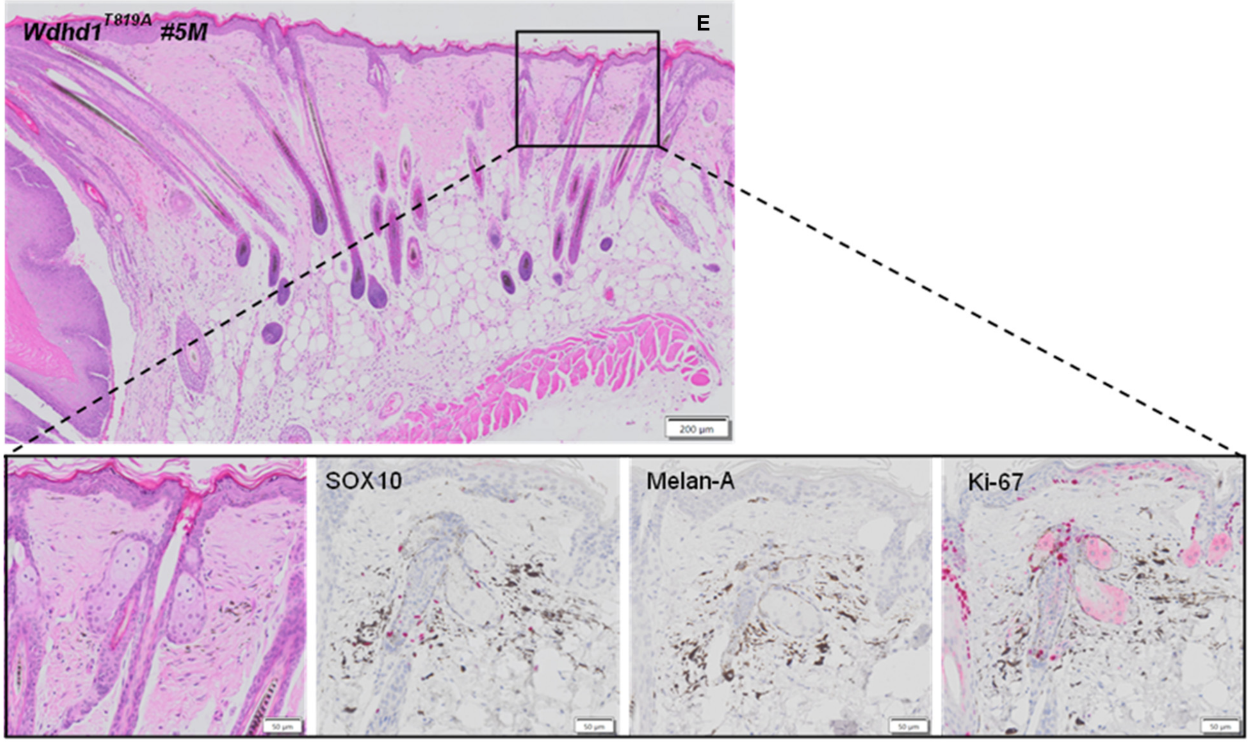

Supplementary Figure 8. Hematoxylin and Eosin (H&E) stain and immunohistochemical (IHC) stain for mice skin tumors

(**A–E**) H&E staining and immunohistochemistry (IHC) for SOX10, Melan-A, and Ki-67 in tumor samples from *Wdhd1*^{T819A} mice (#1–5), with analysis focused on pigmented areas. For antigen retrieval, 4 μm-thick sections for SOX10 and Melan-A were incubated in Tris-EDTA buffer (pH 9.0), while Ki-67 sections were immersed in 10 mM citrate buffer with 0.05% Tween 20 (pH 6.0). All slides were boiled in a microwave at high power for 3 minutes and maintained at 95 °C in a steamer for 15 minutes. After cooling, sections were blocked with PBS containing normal goat serum for 15 minutes, followed by quenching of endogenous peroxidase activity using 1% hydrogen peroxide in PBS for 15 minutes. Slides were then incubated overnight at 4 °C with the following primary antibodies: rabbit anti-SOX10 (Abcam, ab227680; 1:100), rabbit anti-Melan-A (Proteintech, 18472-1-AP; 1:4000), and rabbit anti-Ki-67 (Abcam, ab15580; 1:1000). The next day, sections were incubated with ImmPRESS-AP Polymer Reagent for 30–60 minutes at room temperature, then developed using the ImmPACT Vector Red AP substrate kit (Vectorlabs, SK-5105) for 4 minutes (SOX10), 8 minutes (Melan-A), or 3 minutes (Ki-67). Nuclei were

counterstained with Mayer's hematoxylin for 3 minutes. H&E and IHC images represent non-consecutive serial sections. M: male; F: female. Scale bars: top panels, 200 μm; bottom panels, 50 μm.
