# Supplementary material for: And-1 coordinates with polymerase δ to regulate nucleotide excision repair and UVB-induced skin tumorigenesis
Source: Nat Commun. 2025 Oct 21;16:9313. doi: 10.1038/s41467-025-64380-4 (PMC12540712; doi:10.1038/s41467-025-64380-4)
Supplement: Supplementary file 2 — Description of Additional Supplementary Files [file 41467_2025_64380_MOESM2_ESM.pdf]

Description of Additional Supplementary Files

Supplementary Data 1

This file contains the Mass spec data of And-1 interact proteins

Supplementary Data 2

This file contains the DNA oligos sequences used in this study

Supplementary Data 3

This file contains the histological analysis of all skin tumors from both the *Wdhd1*^{T819A} group (mouse #1-5) and *Wdhd1* WT group (mouse#2)
