# Supplementary material for: And-1 coordinates with polymerase δ to regulate nucleotide excision repair and UVB-induced skin tumorigenesis
Source: Nat Commun. 2025 Oct 21;16:9313. doi: 10.1038/s41467-025-64380-4 (PMC12540712; doi:10.1038/s41467-025-64380-4)
Supplement: Supplementary file 6 — Reporting Summary [file 41467_2025_64380_MOESM6_ESM.pdf]

nature portfolio

Corresponding author(s): Wenge Zhu

Last updated by author(s): Sep 1, 2025

Reporting Summary

Nature Portfolio wishes to improve the reproducibility of the work that we publish. This form provides structure for consistency and transparency in reporting. For further information on Nature Portfolio policies, see our Editorial Policies and the Editorial Policy Checklist.

Statistics

For all statistical analyses, confirm that the following items are present in the figure legend, table legend, main text, or Methods section.

n/a	Confirmed	
☐	☒	The exact sample size (*n*) for each experimental group/condition, given as a discrete number and unit of measurement
☐	☒	A statement on whether measurements were taken from distinct samples or whether the same sample was measured repeatedly
☐	☒	The statistical test(s) used AND whether they are one- or two-sided<br>*Only common tests should be described solely by name; describe more complex techniques in the Methods section.*
☒	☐	A description of all covariates tested
☒	☐	A description of any assumptions or corrections, such as tests of normality and adjustment for multiple comparisons
☐	☒	A full description of the statistical parameters including central tendency (e.g. means) or other basic estimates (e.g. regression coefficient) AND variation (e.g. standard deviation) or associated estimates of uncertainty (e.g. confidence intervals)
☐	☒	For null hypothesis testing, the test statistic (e.g. *F*, *t*, *r*) with confidence intervals, effect sizes, degrees of freedom and *P* value noted<br>*Give P values as exact values whenever suitable.*
☒	☐	For Bayesian analysis, information on the choice of priors and Markov chain Monte Carlo settings
☒	☐	For hierarchical and complex designs, identification of the appropriate level for tests and full reporting of outcomes
☒	☐	Estimates of effect sizes (e.g. Cohen's *d*, Pearson's *r*), indicating how they were calculated

Our web collection on statistics for biologists contains articles on many of the points above.

Software and code

Policy information about availability of computer code

Data collection	Western Blot: Li-cor imager<br>Immunofluorescence: Evos m5000 imaging microscope<br>Plate reader: SpectraMax iD3 Multi-Mode Microplate Readers<br>MS:Thermo Scientific Easy nLC-1000 system coupled with Orbitrap Fusion Lumas Tribrid Mass Spectrometer
Data analysis	Statistical analysis: Graph Pad Prism 9.0 version<br>Western blot analysis: Image Studio Lite Ver 5.2

For manuscripts utilizing custom algorithms or software that are central to the research but not yet described in published literature, software must be made available to editors and reviewers. We strongly encourage code deposition in a community repository (e.g. GitHub). See the Nature Portfolio guidelines for submitting code & software for further information.

Data

Policy information about availability of data

All manuscripts must include a data availability statement. This statement should provide the following information, where applicable:

- Accession codes, unique identifiers, or web links for publicly available datasets
- A description of any restrictions on data availability
- For clinical datasets or third party data, please ensure that the statement adheres to our policy

Mass spectrometry data have been deposited in the ProteomeXchange via the PRIDE partner repository and are associated with the accession number PXD052800 (https://www.ebi.ac.uk/pride/archive/projects/PXD052800). All remaining data can be found in the Article, Supplementary, and Source Data files. Source data are provided in this paper.

Research involving human participants, their data, or biological material

Policy information about studies with human participants or human data. See also policy information about sex, gender (identity/presentation), and sexual orientation and race, ethnicity and racism.

Reporting on sex and gender	N/A
Reporting on race, ethnicity, or other socially relevant groupings	N/A
Population characteristics	N/A
Recruitment	N/A
Ethics oversight	N/A

Note that full information on the approval of the study protocol must also be provided in the manuscript.

Field-specific reporting

Please select the one below that is the best fit for your research. If you are not sure, read the appropriate sections before making your selection.

☒ Life sciences ☐ Behavioural & social sciences ☐ Ecological, evolutionary & environmental sciences

For a reference copy of the document with all sections, see nature.com/documents/nr-reporting-summary-flat.pdf

Life sciences study design

All studies must disclose on these points even when the disclosure is negative.

Sample size	The sample size was determined considering the variations and mean values of the samples, or based on previous observations or a standard protocol in the field. Sample size for each experiment is indicated in the figures or corresponding figure legends.
Data exclusions	No data was excluded from analyses.
Replication	All biological replicates are obtained from biologically independent experiments. All attempts at replication were successful. The experiment numbers are indicated in the figure legends.
Randomization	Samples/mice/participants were randomized allocated into control and experimental groups.
Blinding	The investigators were blinded to group allocation during data collection.

Reporting for specific materials, systems and methods

We require information from authors about some types of materials, experimental systems and methods used in many studies. Here, indicate whether each material, system or method listed is relevant to your study. If you are not sure if a list item applies to your research, read the appropriate section before selecting a response.

Materials & experimental systems

n/a	Involved in the study	
☐	☒	Antibodies
☐	☒	Eukaryotic cell lines
☒	☐	Palaeontology and archaeology
☐	☒	Animals and other organisms
☒	☐	Clinical data
☒	☐	Dual use research of concern
☒	☐	Plants

Methods

n/a	Involved in the study	
☒	☐	ChIP-seq
☒	☐	Flow cytometry
☒	☐	MRI-based neuroimaging

Antibodies

Antibodies used	Anti-β-Actin (CST #3700; 1:5000);<br>Anti-GAPDH (CST #2118; 1:5000);<br>Anti-FLAG-tag (CST #14793; 1:1000);<br>Anti-V5-tag (CST #13202; 1:1000);<br>Anti-Histone H3 (CST #9715; 1:1000);<br>Anti-p125 (sc-17776; 1:1000);<br>Anti-PCNA (sc-56; 1:1000);<br>Anti-p66 (#21935-1-AP; 1:1000);<br>Anti-DDB1 (A14242; 1:1000);<br>Anti-CPDs (CAC-NM-DND-001; 1:1000);<br>Anti-6-4PPs (CAC-NM-DND-002; 1:1000);
Validation	All the antibodies listed above were validated in western blotting.<br>All commercial antibodies have been validated by the manufacturers.

Eukaryotic cell lines

Policy information about cell lines and Sex and Gender in Research

Cell line source(s)	HEK-293T (ATCC, CRL-11268)<br>HEKa (ATCC, PCS-200-011)<br>Hs27 (ATCC, CRL-1634)
Authentication	All cell lines were not authenticated beyond inspection based on morphological criteria
Mycoplasma contamination	The cell lines are routinely tested using mycoplasma detection kit. All cell lines were tested negative for mycoplasma.
Commonly misidentified lines<br>(See ICLAC register)	No commonly misidentified cell lines were used.

Animals and other research organisms

Policy information about studies involving animals; ARRIVE guidelines recommended for reporting animal research, and Sex and Gender in Research

Laboratory animals	C57BL/6J Strain #:000664
Wild animals	This study did not involve wild animals
Reporting on sex	Female C57BL/6J mice were employed
Field-collected samples	This study did not involve field-collected samples
Ethics oversight	Animal procedures were performed according to a protocol approved by the Institutional Animal Care and Use Committee (IACUC) at George Washington University

Note that full information on the approval of the study protocol must also be provided in the manuscript.

Plants

Seed stocks	N/A
Novel plant genotypes	N/A
Authentication	N/A
